# Time-calibrated molecular phylogeny of pteropods

**DOI:** 10.1371/journal.pone.0177325

**Published:** 2017-06-12

**Authors:** Alice K. Burridge, Christine Hörnlein, Arie W. Janssen, Martin Hughes, Stephanie L. Bush, Ferdinand Marlétaz, Rebeca Gasca, Annelies C. Pierrot-Bults, Ellinor Michel, Jonathan A. Todd, Jeremy R. Young, Karen J. Osborn, Steph B. J. Menken, Katja T. C. A. Peijnenburg

**Affiliations:** 1 Naturalis Biodiversity Center, Leiden, The Netherlands; 2 Institute for Biodiversity and Ecosystem Dynamics (IBED), University of Amsterdam, Amsterdam, The Netherlands; 3 Koninklijk Nederlands Instituut voor Onderzoek der Zee (NIOZ), Yerseke, The Netherlands; 4 Natural History Museum (NHM), Cromwell Road, London, United Kingdom; 5 Smithsonian Institution National Museum of Natural History, Washington DC, United States of America; 6 Monterey Bay Aquarium Research Institute (MBARI), Moss Landing, California, United States of America; 7 Molecular Genetics Unit, Okinawa Institute of Science and Technology, Onna-son, Japan; 8 El Colegio de la Frontera Sur (ECOSUR), Unidad Chetumal, Quintana Roo, Chetumal, Mexico; 9 Department of Earth Sciences, University College London, London, United Kingdom; University of California, UNITED STATES

## Abstract

Pteropods are a widespread group of holoplanktonic gastropod molluscs and are uniquely suitable for study of long-term evolutionary processes in the open ocean because they are the only living metazoan plankton with a good fossil record. Pteropods have been proposed as bioindicators to monitor the impacts of ocean acidification and in consequence have attracted considerable research interest, however, a robust evolutionary framework for the group is still lacking. Here we reconstruct their phylogenetic relationships and examine the evolutionary history of pteropods based on combined analyses of Cytochrome Oxidase I, 28S, and 18S ribosomal rRNA sequences and a molecular clock calibrated using fossils and the estimated timing of the formation of the Isthmus of Panama. Euthecosomes with uncoiled shells were monophyletic with *Creseis* as the earliest diverging lineage, estimated at 41–38 million years ago (mya). The coiled euthecosomes (*Limacina*, *Heliconoides*, *Thielea*) were not monophyletic contrary to the accepted morphology-based taxonomy; however, due to their high rate heterogeneity no firm conclusions can be drawn. We found strong support for monophyly of most euthecosome genera, but *Clio* appeared as a polyphyletic group, and *Diacavolinia* grouped within *Cavolinia*, making the latter genus paraphyletic. The highest evolutionary rates were observed in *Heliconoides inflatus* and *Limacina bulimoides* for both 28S and 18S partitions. Using a fossil-calibrated phylogeny that sets the first occurrence of coiled euthecosomes at 79–66 mya, we estimate that uncoiled euthecosomes evolved 51–42 mya and that most extant uncoiled genera originated 40–15 mya. These findings are congruent with a molecular clock analysis using the Isthmus of Panama formation as an independent calibration. Although not all phylogenetic relationships could be resolved based on three molecular markers, this study provides a useful resource to study pteropod diversity and provides general insight into the processes that generate and maintain their diversity in the open ocean.

## Introduction

Pteropods are a group of holoplanktonic heterobranch gastropod molluscs [1] that are widespread and abundant in the marine zooplankton (e.g. [2,3]). They have been proposed as bioindicators to monitor the effects of ocean acidification because their aragonite shells are exceptionally vulnerable to rising levels of CO_2_ in the global ocean (e.g. [4–7]). It is expected that anthropogenic carbon input into the ocean may affect marine life more severely than in the past, because it is happening much faster than, for instance, at the Paleocene-Eocene thermal maximum (PETM) ~56 million years ago (mya) [8–10]. During the PETM, massive amounts of carbon were released into the atmosphere and ocean, leading to ocean acidification and warming, and the ocean’s calcite saturation depth shoaled by at least 2 km within 2000 years, a situation that persisted for tens of thousands of years [9,11]. This resulted in major shifts in marine planktonic communities, including foraminifers and calcareous phytoplankton, and a major extinction of benthic foraminifers, but probably not of pteropods [8,10,12–17].

Pteropods comprise the orders Thecosomata and Gymnosomata [18]. These groups are ecologically distinct: the thecosomes produce mucus webs to feed on microplankton, whilst gymnosomes are active predators that often feed on thecosomes [19,20]. The thecosomes are further divided into the suborders Euthecosomata and Pseudothecosomata [21]. The euthecosomes have aragonite shells throughout their lives while the pseudothecosomes have coiled shells or a semi-soft pseudoconch with an aragonite shell only during the larval stage [20,22]. Within the euthecosomes, the superfamily Limacinoidea has coiled shells, and the superfamily Cavolinioidea has uncoiled shells. Gymnosomes have only larval shells, which they shed during metamorphosis into adults [23]. Most pteropod species are epipelagic, but some species are meso- or bathypelagic (e.g. *Clio andreae*, *C*. *chaptalii*, *C*. *piatkowskii*, *C*. *polita*, *C*. *recurva*, *Thielea helicoides*, and *Peracle bispinosa*) [20,24].

The taxonomy of thecosomes, and especially that of euthecosomes, has been revised frequently and remains disputed, especially at the (super)family level. The most widely used taxonomy (as accepted by [25]) was proposed by [26]. In this classification, the Limacinidae (with extant genera *Heliconoides*, *Limacina*, and *Thielea*) are the only family within the superfamily Limacinoidea, whereas most other classifications included the Limacinidae in the Cavolinioidea [27,28]. The superfamily Cavolinioidea contains the extant families Cavoliniidae (*Cavolinia*, *Diacavolinia*, *Diacria*), Cliidae (*Clio*), Creseidae (*Creseis*, *Hyalocylis*, *Styliola*), and Cuvierinidae (*Cuvierina*) [26]. In other studies, Cavolinioidea has been ranked as the family Cavoliniidae with three subfamilies: Cavoliniinae (*Cavolinia*, *Diacavolinia*, *Diacria*), Clionae (*Clio*, *Creseis*, *Hyalocylis*, *Styliola*), and Cuvierininae (*Cuvierina*) [27–29]. Finally, according to [30–32] there are two families within the Cavolinioidea: Creseidae (*Creseis*, *Hyalocylis*, *Styliola*) and Cavoliniidae, comprising the subfamilies Cavoliniinae (*Cavolinia*, *Diacavolinia*, *Diacria*, *Clio*) and Cuvierininae (*Cuvierina*).

Several recent studies have tested pteropod taxonomic hypotheses using genetic data. Pteropods were confirmed as a monophyletic group within the Opisthobranchia based on a sampling of ten euthecosome and three gymnosome taxa using the three molecular markers Cytochrome oxidase subunit I (COI) mitochondrial DNA, nuclear 28S, and 18S rRNA (28S and 18S respectively) [33]. Anaspidea was proposed as the most likely sister group of pteropods [33], and this was confirmed by phylogenomic analyses of gastropods [34]. Within the pteropods, the uncoiled euthecosomes and gymnosomes were recognized as monophyletic groups in molecular phylogenetic analyses [33,35]. [36] constructed phylogenies based on COI sequences of 30 pteropod species. They demonstrated that phylogenetic relationships of pteropods above the species level could not be reliably established using COI as the only molecular marker because of the high rate heterogeneity and limited phylogenetic signal of this marker. [35] proposed an evolutionary scenario for thecosome pteropods based on a cladistic analysis of morphological data of extant species and separate phylogenetic analyses of COI and 28S genes, with pseudo- and euthecosomes splitting first, and coiled euthecosomes as a paraphyletic group from which uncoiled euthecosomes evolved. Based solely on 28S, both [33] and [35] reported *Creseis* as the earliest diverging member of the uncoiled euthecosomes.

Some euthecosome taxa have been studied in more detail using molecular methods. Based on COI sequences, [37] found that Arctic and Antarctic *Limacina helicina* populations represented genetically distinct species. The Creseidae were studied by [38], who found monophyly of the genera *Creseis*, *Hyalocylis*, and *Styliola* based on COI data. *Cuvierina* and *Diacavolinia* have been studied using integrative taxonomic approaches combining COI and/or 28S data, morphological analyses of shells, and/or geographic information [39–41]. Six *Cuvierina* morphotypes with distinct geographic distributions were distinguished based on geometric morphometric analyses of shell shapes [40], one of which was described as a new species [42]. [41] found evidence for a reduction in the number of described *Diacavolinia* species from 24 to a maximal estimate of 13 species based on integrative taxonomic analyses using recently collected specimens as well as museum specimens, including type material.

Pteropods have an extensive fossil record that largely consists of euthecosome shells and larval gymnosome shells (e.g. [22,43]). Because their fragile shells do not survive significant transport, they are rarely reworked from one sedimentary unit into another, which makes them reliable fossils for biostratigraphic purposes (e.g. [44]). Combining their fossil record with molecular methods for phylogenetic inference can improve our understanding of the evolutionary history of pteropods, and provides a framework for assessing past and present responses to global change.

Vicariance events in the global ocean, such as the Terminal Tethyan Event (TTE) and uplift of the Isthmus of Panama (IOP), have had profound effects on the evolution of marine organisms (e.g. [45–49]). These include reduced connectivity by changes in ocean circulation and shifts in centers of marine diversity. However, the onset of these land barriers has been complex and the timing of events is widely debated, with the IOP formation most commonly estimated at ~3 mya (e.g. [47,49–51]). Hence, a better understanding of when these events occurred has important implications for using vicariance events for calibrating molecular clocks for different species as well as for cross-validating fossil-calibrated molecular clocks. [36] demonstrated that several pteropod species had interspecific levels of sequence divergence between lineages from different ocean basins. Divergent clades for Atlantic and Pacific sister taxa would allow the use of the IOP as a molecular clock calibration.

Here we shed new light on the evolutionary history of pteropods by performing a combined analysis of three molecular markers: COI, 28S and 18S genes. We include 55 pteropod species spanning the diversity of the group and sampled from each ocean basin, including molecular data of seven additional euthecosome, two pseudothecosome, and five gymnosome species relative to prior studies. We use fossil evidence and the dating of the formation of the Isthmus of Panama to reconstruct the evolutionary history of pteropods, thus enabling comparisons between different calibration methods. Our aims are to (1) examine the phylogenetic relationships of pteropods sampled from the global ocean based on COI, 28S, and 18S sequence data and (2) reconstruct the evolutionary history of pteropods using a molecular clock approach.

## Materials and methods

### Sampling and specimens

**Fig 1:**
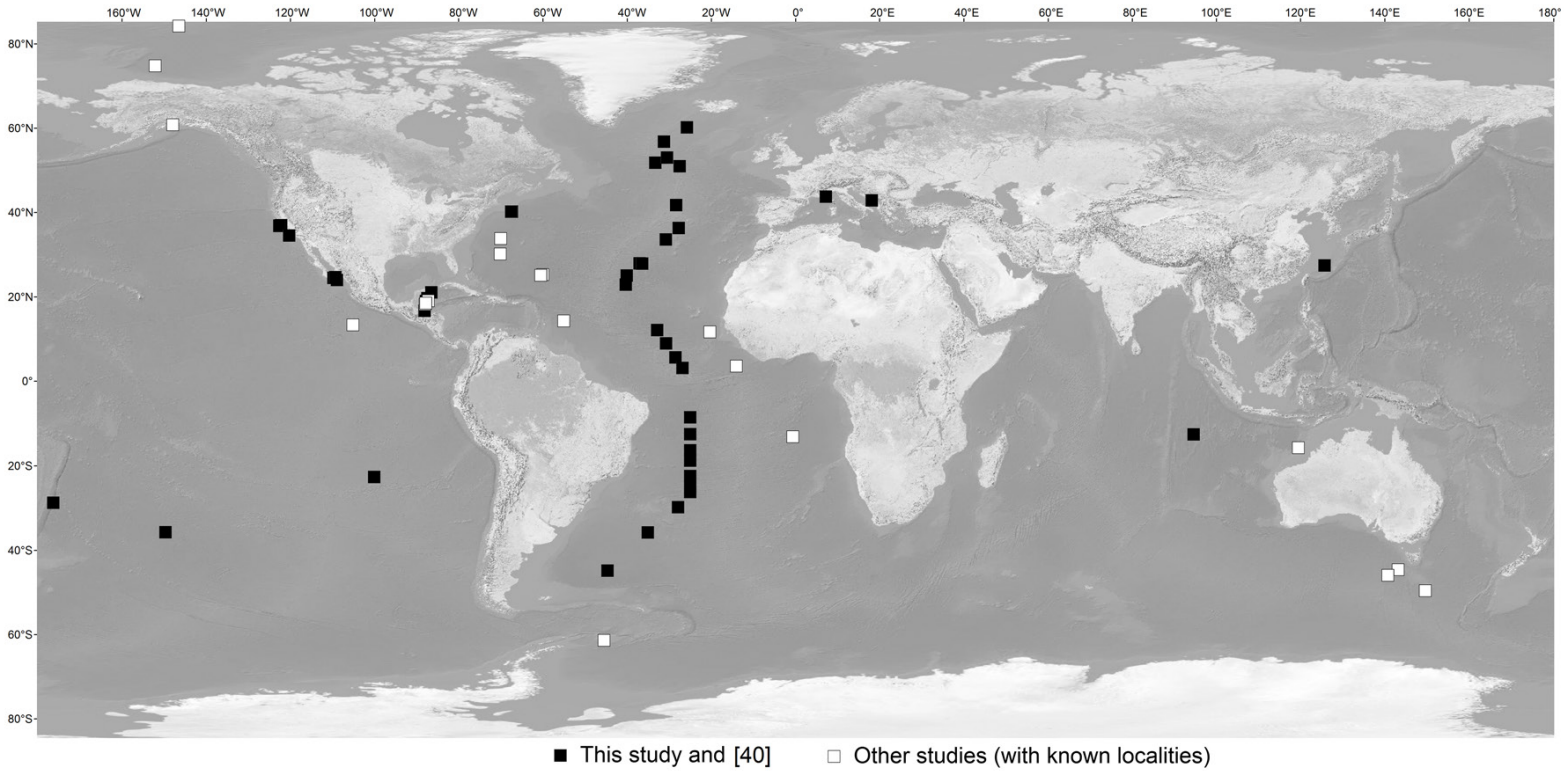
Overview of sampling locations. Black squares indicate sampling locations of new specimens in this study and white squares indicate all known locations of specimens used from other studies. Sampling locations from [33], [35], and [37] are not indicated because no exact localities were given (*see* S1 Table).

A total of 55 pteropod species were included in this study (treating subspecies as separate species here and subsequently; S1 Table). Of these, 27 species are uncoiled euthecosomes and eight are coiled euthecosomes, together representing up to 56% of all currently recognised extant euthecosome species, as well as all eight uncoiled and all three coiled genera. In addition, eight pseudothecosome species are included (35% of all species), representing all five genera. Twelve gymnosome species (23% of all species) are included, representing eight of the 19 genera. Pteropods were collected at a total of 90 stations: 58 in the Atlantic Ocean (40 species), 14 in the Pacific Ocean (14 species), 10 in the Indian Ocean (10 species), 6 in the Southern Ocean (7 species), and 2 in the Arctic Ocean (2 species) (Fig 1; S1 Table). New samples for this study were collected from the Atlantic Ocean and Caribbean Sea during the AMT18, AMT22, ECO-CH-Z, and MAR-ECO expeditions between 2004 and 2012, and from net tows, remotely operated vehicles and bluewater SCUBA dives in the Northwest Atlantic Ocean, Northeast Pacific Ocean, and Gulf of California in 2012 and 2013. Permits were available for sampling in the Gulf of California (permits DAPA/2/080211/00217 by Comisión Nacional de Acuacultura y Pesca, CTC/001340 by La Secretaria de Relacione Exteriores, and H00/INAPESCA/DGIPPN/831 by Secretaria de Agricultura, Ganaderia, Desarrollo Rural, Pesca Y Alimentacion) and the NE Pacific Ocean (California Department of Fish and Wildlife Scientific Collecting Permit SC-2316). No permits were required for other plankton collections, and the work did not involve any endangered or protected species.

The majority of pteropod specimens were collected from the epi- to upper mesopelagic layer (500–0 m depth) using different types of plankton nets. Sampling details can be obtained from shipboard reports available online (e.g. [52] for AMT cruises, [53] for the MAR-ECO cruise, and [54–56]). Pteropod sequences from remaining locations were available from [33–40]. Sampling information for all specimens in this study, including collection dates, geographical coordinates, cruise and station numbers, if reported in previous work, are collated in S1 Table. Images of newly collected specimens are deposited in the Dryad digital repository [57].

### DNA extraction, amplification, and sequencing

Genomic DNA was extracted from muscle tissue or entire individuals (when smaller than 3 mm) using the DNeasy blood & tissue Kit (Qiagen). Alternatively, genomic DNA from specimens collected in 2012 and 2013 from the Northwest Atlantic and Northeast Pacific oceans and the Gulf of California was extracted using an AutoGenprep965 high throughput system (AutoGen Inc., Holliston, USA) using manufacturer protocols.

A fragment of ~600 basepairs (bp) of COI was amplified using the primers LCO-1490 (5′-GGTCAACAAATCATAAAGATATTGG-3′) and HCO-2198 (5′-TAAACTTCAGGGTGACCAAAAAATCA-3′) [58]. A fragment of ~1000bp of 28S was amplified using primers C1-F (5’-ACCCGCTGAATTTAAGCAT-3’) [59] and D3-R (5’-GACGATCGATTTGCACGTCA-3’) [60]. The 18S region of ~1800bp was amplified by two overlapping fragments for most samples (overlap of ~500bp). The two fragments were amplified using the primer pairs A1-F (5’-CTGGTTGATCCTGCCAGTCATATGC-3’) [60] and 18S-KP-R (5’-TTCCCGTGTTGAGTCAAATTAAG-3’) (this study) and 18S-KP-F (5’-TGGAGGGCAAGTCTGGTG-3’) (this study) and 1800R (5’-GATCCTTCCGCAGGTTCACCTACG-3’) [60].

PCR amplification of COI was performed using 25μL volumes containing 15.7μl ddH_2_O, 2.5μl 10xPCR buffer, 2.5μl DNTPs (1mM each), 1μl MgCl_2_, 0.2μl BSA (10mg/ml), 0.2μl of each primer, 0.2μl Super*Taq*, and 2.5μl template DNA, or using illustraTM puReTaq Ready-To-Go PCR Beads (GE Healthcare) requiring 20μl ddH_2_O, 1μl of each primer, and 3μl template DNA. The PCR cycling steps were an initial denaturation step of 94°C for 180s followed by 35 cycles of 94°C for 45s, 45°C for 60s, and 72°C for 120s, and a final extension of 72°C for 600s. Alternatively, for specimens collected in 2012 and 2013 from the Northwest Atlantic and Northeast Pacific oceans, PCR amplification of COI was performed in 10μL volumes containing 5.95μL ddH_2_O, 0.6μL MgCl_2_, 0.5μL dNTPs, 0.25μL BSA, 0.3μL of each primer, 0.1μL Biolase DNA polymerase (BioLine, Taunton MA), 1μL manufacturer-provided buffer, and 1μL template DNA, with an initial denaturation at 95°C for 300s, 40 cycles of 95°C for 30s, 48°C for 30s, and 72°C for 45s, with a final extension at 72°C for 300s. For most specimens, PCR amplification of both 18S and 28S was performed in 20μL volumes containing 8.8μL ddH_2_O, 4μL 5x Phire buffer (Phire), 2μL dNTPs (1mM each), 1.4μL 100% DMSO, 0.2μL BSA (10mg/ml), 0.5μL of each primer (18S: A1- and 1800R or A1-F and KP-R and KP-F and 1800-R, 28S: C1-F and D3-R), 0.2μL Hot Start *Taq* (Phire) and 2.4μL template DNA. Alternatively, 25 μL volumes were used, containing 7μL ddH_2_O, 2.5μL BSA, 1μL of each primer, 12.5μL Amplitaq Gold Fast *Taq* (Life Technologies, Grand Island, NY, USA), and 1μL template DNA. PCR cycling steps were an initial denaturation step of 98°C for 30s, 35 cycles of 98°C for 5s, 48°C for 5s and 72°C for 20s, a final extension step of 72°C for 60s, and a cooling step of 4°C for 180s. Bands were checked on agarose gel followed by a purification step of the PCR product using the QIAquick gel extraction kit (Qiagen, Germany). After a final cycle sequencing reaction using ABI BigDye Terminator cycle sequencing kit vol. 1.1 (Life Technologies, USA) according to the manufactures protocol, PCR products were sequenced by Macrogen Europe, The Netherlands, the Laboratories of Analytical Biology at the Smithsonian National Museum of Natural History, USA, or the Monterey Bay Aquarium Research Institute, USA.

### Sequence alignments

We aimed to include as many complete COI, 28S, and 18S fragments for available taxa as possible in our sequence alignments. The data collected in this study was complemented by sequences from prior studies (S1 Table). For some specimens we combined partitions from different specimens of the same species and the same ocean for the multi-gene alignments (S1 Table). For inclusion in multi-gene alignments, sequences of at least two of the three markers per taxon had to be available.

We obtained a total of 53 COI, 53 28S, and 46 18S sequences in this study, and used an additional 65 COI, 35 28S, and six 18S sequences from prior studies for various purposes (S1 Table). We included two outgroup species sequenced by [33]in all alignments: the large sea slug *Aplysia californica* (Anaspidea), and the shelled gastropod *Bulla striata* (Cephalaspidea). These species were chosen as outgroups because *Aplysia californica* is a representative of the most likely sister group of the Pteropoda, and *Bulla striata* represents a possible sister group to Anaspidea + Pteropoda [33].

Nucleotide sequences of COI, 28S, and 18S were examined in MEGA 6 [61] and aligned using MAFFT v7 [62]. The amino acid alignment of COI (AACOI) was aligned using the MUSCLE algorithm [63] in MEGA 6. All sites were used for COI as well as for the AACOI alignment. For 28S and 18S, sites with a coverage of <80% in the alignments were removed using trimAl [64] in Phylemon 2.0 [65]. This resulted in final alignment lengths of 656bp or 218 amino acids for COI, 941bp for 28S, and 1683bp for 18S. To estimate evolutionary relationships among taxa based on single-gene trees, we used an alignment of N = 117 sequences representing 53 pteropod species for COI, N = 87 sequences (44 species) for 28S, and N = 52 sequences (28 species) for 18S. For the multi-gene phylogeny, we used AACOI, 28S, and 18S partitions comprising a subset of 78 specimens to have a more even taxon sampling across groups (42 pteropod species; a maximum of two taxa per species per ocean). The 28S partition was complete and for the other partitions, 77 AACOI sequences were available (only missing for *Limacina bulimoides*), as well as 52 18S sequences (representing all euthecosome genera except *Thielea*, representing the pseudothecosome genera *Corolla* and *Peracle*, but missing for *Cymbulia*, *Gleba*, and *Desmopterus*, and representing six gymnosome genera except *Notobranchaea* and *Schizobrachium*, regarding only the genera included elsewhere in this study). To produce a time-calibrated phylogeny, we used a subset of 46 sequences (40 pteropod species, a maximum of one sequence per species per ocean) consisting of AACOI (N = 45), 28S (N = 46), and 18S (N = 34) partitions, because the Yule tree prior assumes that all taxa are independent (i.e., reproductively isolated) [66].

### Phylogenetic analyses

We first estimated phylogenetic relationships based on single-gene Maximum Likelihood (ML) analyses, followed by multi-gene ML analyses, and Bayesian, time-calibrated analyses. To estimate evolutionary relationships among taxa based on single as well as combined genes, we applied a ML approach [67] in RaxmlGUI 1.3 [68,69]. We tested for the most appropriate model of sequence evolution per gene separately using the Akaike Information Criterion (AIC) in jModelTest 2.1.3 [70] based on 88 models, and this approach always selected the GTR (General Time Reversible) model with a proportion of invariable sites (I) and gamma distributed rate variation among sites (Γ). For the AACOI alignment, we used the MtZoa model of evolution, because it best represents the evolutionary process and reduces systematic bias [71].

For the single-gene ML phylogenies, we applied a ML search followed by a non-parametric bootstrap analysis with 1500, 2000, and 3000 replicates for COI, 28S, and 18S, respectively. For the multi-gene combined ML phylogeny, we used the MtZoa model of evolution for AACOI and GTR+Γ+I for 28S as well as 18S, and applied a ML search followed by 1500 bootstrap replicates. To examine the effect of long-branch taxa on the phylogenetic signal, an additional tree of 75 specimens (40 pteropod species) was generated excluding *Limacina bulimoides* and *Heliconoides inflatus*.

Prior to producing a time-calibrated phylogeny, we independently tested the topology of the dataset in RaxmlGUI using the same methods as described above. We subsequently applied relaxed Bayesian molecular clock analyses with uncorrelated log-normal rates in BEAUti and BEAST 1.81 [72]. Because the MtZoa model of evolution was not available in BEAUti 1.81, we instead used the WAG model [73] for AACOI and GTR+Γ+I for 28S and 18S.

The molecular clock analyses used the Yule Process of speciation [66] and were calibrated using three different methods as summarized in Table 1 (*see also* [72]). In all three methods, the stem node of the pteropods was calibrated using a normally distributed secondary calibration derived from a phylogenomic gastropod study by [34], and the crown node of the euthecosomes was calibrated using a log-normal distribution based on the geological age of the first known euthecosome pteropod, *Heliconoides* sp., from the Late Cretaceous [74]. In the first calibration approach (Method 1), we used the geological ages of the oldest known fossils presumed to belong to the extant euthecosome genera *Hyalocylis*, *Diacria*, *Cavolinia*, *Cuvierina*, *Creseis*, and *Limacina* as log-normally distributed crown calibrations (Table 1) [43,74–81]. These fossils were of Eocene, Miocene, or Pliocene age (Table 1). In the second approach (Method 2), we set these crown calibrations as genus stem calibrations instead, excluding the *Limacina* calibration. The significant genetic differentiation between Atlantic and Pacific populations of the euthecosomes *Clio pyramidata* and *Hyalocylis striata*, and the gymnosome *Cliopsis krohni* was likely a consequence of the emergence of the Isthmus of Panama (IOP). Hence, in a third calibration approach (Method 3) we used the IOP as a normally distributed prior with a mean of 3.1 mya and a standard deviation of 1 million years, accounting for the debated IOP timing [49]. For all approaches, two MCMC chains were run of 10^8^ generations each. We sampled trees and log-likelihood values at 10^4^-generation intervals. Sets of trees obtained during independent runs were combined in LogCombiner 1.8.1 [72], and the maximum clade credibility trees were selected using TreeAnnotator 1.8.1 [72]. To cross-validate calibrations and derive independent ages of these nodes, we performed additional runs of 10^8^ generations while leaving out one calibration at a time.

**Table 1:** Overview of molecular clock calibration settings using three different methods.

Calibrated node	Calibration type	Calibration method			Calibration age		Prior settings					References
		1	2	3	mya	Epoch: age	Prior dist.	Offset	Mean	Log (Stdev)	Stdev	
*Clio pyramidata* (excl. *C*. *p*. *antarctica*)	IOP: Atlantic—Pacific			Crown	3.1	Pliocene: Piacenzian	Normal		3.1		1	[49]
*Hyalocylis striata*	IOP: Atlantic—Pacific			Crown	3.1	Pliocene: Piacenzian	Normal		3.1		1	[49]
*Cliopsis krohni*	IOP: Atlantic—Pacific			Crown	3.1	Pliocene: Piacenzian	Normal		3.1		1	[49]
*Hyalocylis*	Fossil: *Hyalocylis marginata*	Crown	Stem		3.6–2.6	Pliocene: Piacenzian	Log-normal	2.6	0.8	0.7		[43]
*Diacria*	Fossil: *Diacria sangiorgii*	Crown	Stem		11.6–7.2	Miocene: Tortonian	Log-normal	7.2	2.5	0.7		[78]
*Cavolinia—Diacavolinia*	Fossil: *Cavolinia zamboninii*	Crown	Stem		16–13.8	Miocene: Langhian	Log-normal	13.8	1.5	0.7		[77]
*Cuvierina*	Fossil: *Cuvierina torpedo*	Crown	Stem		23–20.4	Miocene: Aquitanian	Log-normal	20.4	1.5	0.7		[76,80]
*Creseis*	Fossil: *Creseis corpulenta*	Crown	Stem		38–41	Eocene: Bartonian	Log-normal	38	1.5	0.7		[75,81]
*Limacina*	Fossil: *Limacina gormani*	Crown			56–47.8	Eocene: M-L Ypresian	Log-normal	48	8	0.7		[79,81]
Euthecosomata	Fossil: *Heliconoides* sp.	Crown	Crown	Crown	79–66	Late Cretaceous: Late Campanian/Maastrichtian	Log-normal	65.5	3	0.7		[74]
Pteropods vs. outgroup taxa	Secondary: *Clione antarctica* vs. *Aplysia californica*	Stem	Stem	Stem	170–90		Normal		130		14	[34]

Method 1 uses fossil ages as crown calibrations, Method 2 uses fossil ages as stem calibrations, and Method 3 uses the formation of the Isthmus of Panama (IOP) as crown calibrations. mya = million years ago; Prior dist. = Prior distribution; Stdev = Standard deviation.

## Results

### Sequence alignments

The numbers of indels in the COI, 28S, and 18S sequence alignments vary considerably between euthecosomes, pseudothecosomes, and gymnosomes. No stop codons or frameshift mutations are present in the COI alignment (656bp). The same consecutive three codons are missing for all *Limacina*, *Corolla*, *Cymbulia*, and *Gleba* species. Additional deletions of one or two codons are observed for *Hyalocylis* and *Limacina* species. No insertions or deletions are observed in gymnosome sequences. In the trimmed 28S alignment (941bp), the number of species or genus-specific gaps is highly variable. Within the euthecosomes, *Heliconoides inflatus* sequences have the highest number of gaps (20bp), followed by 6–15bp for *Limacina bulimoides*, *L*. *trochiformis*, all *Cresei*s species, and *Hyalocylis striata*. Within the pseudothecosomes, 15bp are missing for *Desmopterus* sp., followed by 3–7bp for *Cymbulia* sp., *Corolla spectabilis*, and *Peracle reticulata*. Of the gymnosomes, only *Spongiobranchaea australis* has a gap (1bp). In the trimmed 18S alignment (1683bp), a large number of gaps is found for *Limacina bulimoides* (47bp), followed by *Creseis clava* (13bp), and *Heliconoides inflatus* (11bp). Few gaps are present within the pseudothecosome sequences (1–3bp for *Corolla spectabilis* and *Peracle reticulata*). Contrary to COI and 28S alignments of gymnosome taxa, some gaps remain in the 18S alignment of the gymnosomes *Pneumoderma atlantica* (10bp), and 1–2bp for *Pneumodermopsis* sp., *Spongiobranchaea australis*, *Clione limacina antarctica*, and *Thliptodon* sp.

### Individual gene trees

Gene trees recovered from separate ML analyses of COI, AACOI, 28S, and 18S datasets generally recover species and genera with moderate to high bootstrap support (>80%), however, higher-level groupings are unstable and unsupported (S1–S4 Figs). In both COI gene trees (S1 and S2 Figs for nucleotides and amino acids, respectively) the coiled *Heliconoides inflatus* and *Thielea helicoides* group with the uncoiled euthecosomes, rendering both superfamilies of Euthecosomata, uncoiled Cavolinoidea and coiled Limacinoidea, polyphyletic. This could be due to long branches of all *Limacina* taxa (coiled), and *Styliola subula* and *Hyalocylis striata* (uncoiled). Higher-level taxa such as the Euthecosomata, Pseudothecosomata, and Gymnosomata are not significantly supported and their relationships are unresolved based on COI. Supported geographic subclades within species sampled from different ocean basins are recovered for Atlantic and Pacific populations of *Cavolinia uncinata*, *Clio pyramidata*, and *Hyalocylis striata* (euthecosomes), and the gymnosome *Cliopsis krohni* (S1 Fig). In the 28S tree, all genera are recovered with moderate to high bootstrap support, except *Clio* (S3 Fig). There is one well-supported clade with *Clio pyramidata* and *C*. *convexa* (96% bootstrap support), with *C*. *cuspidata* and *C*. *recurva* falling into the basal polytomy. Relationships between different genera are mostly unresolved, but the genus *Cavolinia* is monophyletic only when *Diacavolinia* is included within *Cavolinia*. *Heliconoides inflatus* is on an exceptionally long branch. Similarly, the 18S tree supports most genera but does not resolve any of the higher-level groupings, likely because of extremely long branches of two species, *Heliconoides inflatus* and *Limacina bulimoides* (S4 Fig).

### Multi-gene phylogenies

**Fig 2:**
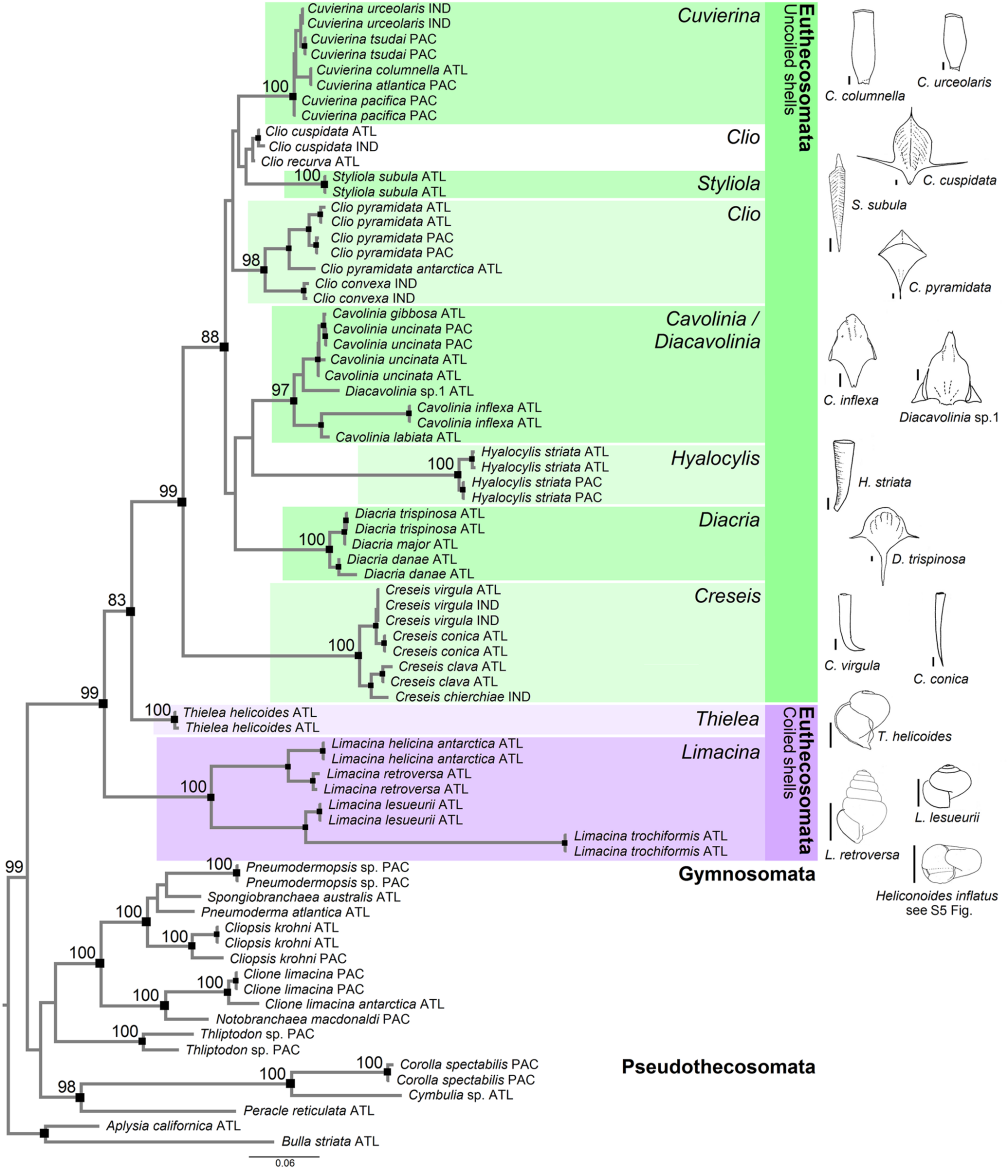
Maximum Likelihood phylogeny of pteropods based on the concatenated dataset of Cytochrome Oxidase I (amino acid alignment) and ribosomal genes 28S and 18S (nucleotide alignments) excluding the long-branch taxa *Heliconoides inflatus* and *Limacina bulimoides*. Black squares represent a bootstrap support of ≥80%, with small, medium and large black squares representing support within genera, of genera, and above genus level, respectively. Supported euthecosome genera are highlighted in coloured boxes, and representative shell shapes of species are drawn on the right, with all scale bars representing 1 mm. Maximum Likelihood phylogeny including the long-branch taxa *Heliconoides inflatus* and *Limacina bulimoides* is shown in S5 Fig. Abbreviations ATL, PAC, and IND denote Atlantic, Pacific, and Indian Ocean origins, respectively, including their sectors in the Southern Ocean.

Combining COI, 28S, and 18S sequences into a single phylogenetic analysis with the three genes as separate partitions (partially) compensates for the effects of rate heterogeneity within specific partitions between taxa and improves phylogenetic resolution. Concatenated datasets both including (S5 Fig) and excluding the long-branch taxa *H*. *inflatus* and *L*. *bulimoides* (Fig 2) have very similar topologies. Both phylogenies recover Pteropoda as a monophyletic group (100% and 99% support, respectively) as well as Euthecosomata (99% in both phylogenies) and Pseudothecosomata (99% and 98%, respectively). However, in both phylogenies the monophyly of Gymnosomata is not supported due to the exclusion of *Thliptodon* from the clade. The phylogenetic relationships between euthecosomes, pseudothecosomes, and gymnosomes are unresolved based on these three genes.

The uncoiled euthecosomes (eight genera, superfamily Cavolinioidea) are a well-supported monophyletic group (99% support) in the analyses that exclude long-branch taxa, and *Creseis* is the sister group to all other genera within this clade (Fig 2). However, relationships between other uncoiled euthecosome genera are unresolved. The coiled euthecosome genera *Thielea* and *Limacina* (superfamily Limacinoidea) are not supported as a clade but represent the earliest diverging branches within Euthecosomata (Fig 2), and the phylogenetic position of *Heliconoides* remains unknown. In the phylogeny with long-branch taxa removed (Fig 2), the bathypelagic coiled species *Thielea helicoides* is the sister group of all uncoiled euthecosomes with moderate bootstrap support (83%).

Most species and genera are recovered as monophyletic groups in multi-gene phylogenies except *Clio* and *Cavolinia* (Fig 2; S5 Fig). *Clio pyramidata*, *C*. *pyramidata antarctica*, and *Clio convexa* represent one well-supported clade and *C*. *cuspidata* and *C*. *recurva* represent another unsupported group with unresolved affinities. *Diacavolinia* sp. groups with *Cavolinia* species rendering the genus *Cavolinia* paraphyletic. Within Pseudothecosomata, the pseudoconch genera *Corolla* and *Cymbulia* group separately (100% bootstrap) from the shelled *Peracle* species. The genus *Thliptodon* does not form a supported clade with other gymnsosome genera that do represent a well-supported monophyletic group (*Clione*, *Cliopsis*, *Notobranchaea*, *Pneumoderma*, *Pneumodermopsis*, and *Spongiobranchaea*). The latter clade consists of two subclades, one comprising *Clione* and *Notobranchaea* (100% support), and the other containing *Cliopsis*, *Pneumoderma*, *Pneumodermopsis*, and *Spongiobranchaea* (100% support, Fig 2; S5 Fig).

### Time-calibrated phylogenies

For molecular clock analyses, we reduced the number of taxa in the concatenated dataset to one taxon per species per ocean basin, and kept the long-branch taxa *Heliconoides inflatus* and *Limacina bulimoides*. We first performed a ML analysis to confirm that tree topologies were identical with previous multi-gene phylogenies. This is clearly the case, and Gymnosomata also represent a well-supported clade in this analysis (S6 Fig).

**Fig 3:**
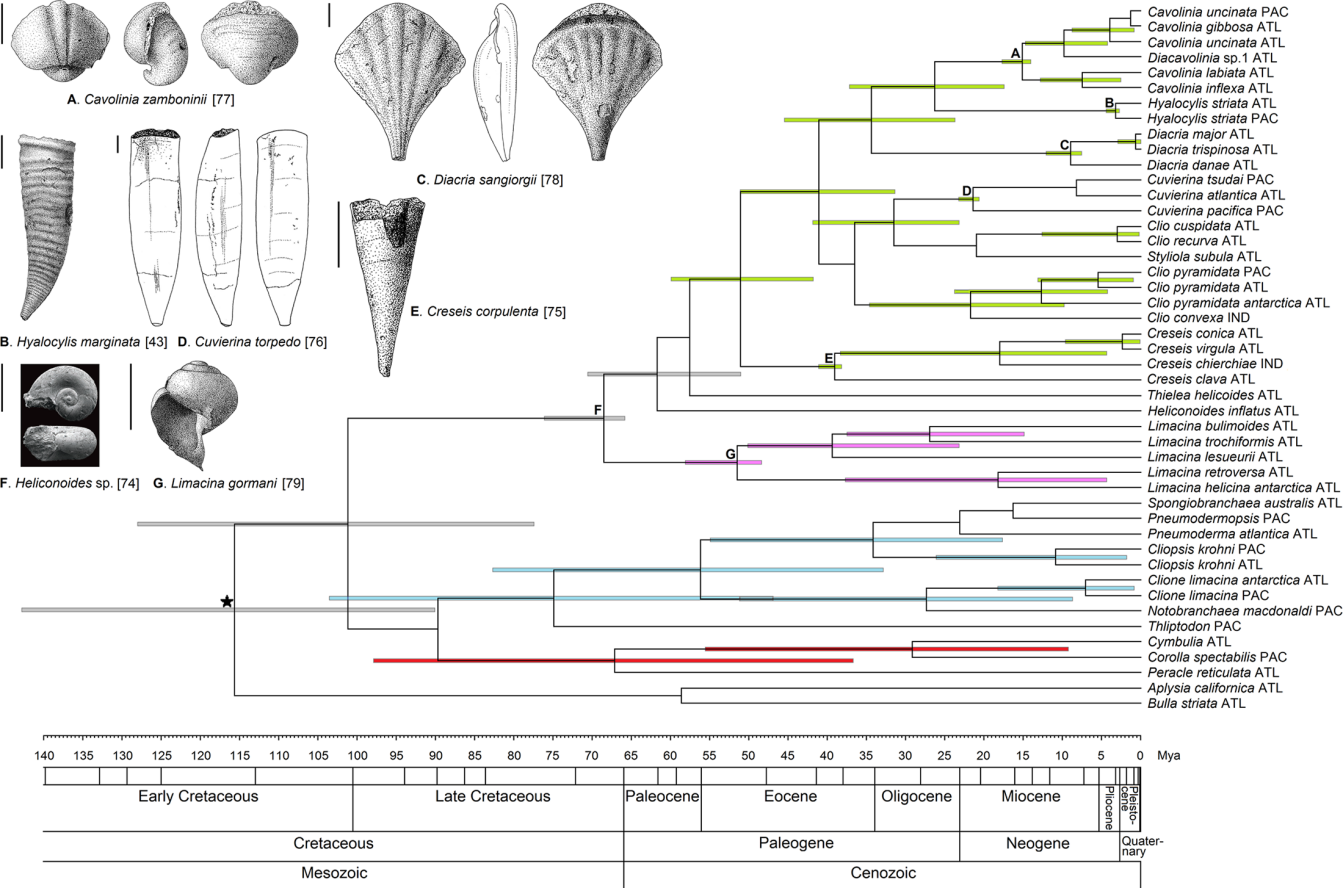
Fossil-calibrated phylogeny of pteropods (46 taxa, maximum one sequence per taxon per ocean) following Method 1 with crown calibrations based on the oldest known fossils of *Hyalocylis*, *Diacria*, *Cavolinia*, *Cuvierinia*, *Creseis*, *Limacina*, and euthecosomes (*see* Table 1). Drawings of fossils used for calibration are shown on the left, with all scale bars representing 1 mm, and calibrations are indicated with letters A to G. Error bars (95%) are shown only for clades with posterior probabilities ≥0.95 in green for uncoiled euthecosomes, purple for coiled euthecosomes, red for pseudothecosomes, and blue for gymnosomes. Abbreviations ATL, PAC, and IND denote Atlantic, Pacific, and Indian Ocean origins, respectively, including their sectors in the Southern Ocean.

The topologies of Bayesian time-calibrated phylogenies based on three approaches are identical and match unconstrained ML analyses (Fig 3; S7 and S8 Figs), for calibration methods 1, 2 and 3, respectively). Posterior probabilities of clade support are high for most nodes (>0.95), including the monophyletic euthecosome, pseudothecosome, and gymnosome clades. Within the uncoiled euthecosomes, significant support is observed for several additional clades compared to the ML phylogenies: the *Clio cuspidata*/*recurva* group, the *Cuvierina* + *Clio cuspidata*/*recurva* + *Styliola* group, the *Cavolinia/Diacavolinia* + *Hyalocylis* group (only for Methods 1 and 3), and the *Cavolinia/Diacavolinia* + *Hyalocylis* + *Diacria* group.

The node ages of pteropod genera derived from molecular clocks are less variable than node ages at higher taxonomic levels. The crown group ages of euthecosome genera based on fossil ages (Method 1) correspond well with the ages based on IOP calibrations (Method 3), and fossil-based stem calibrations (Method 2) generally lead to much younger crown group ages than other calibration methods (Fig 3; Table 2; S7 and S8 Figs). Cross-validation analyses, in which we left out one of the fossil crown calibrations at a time, show that the derived node ages correspond well with the calibrated ages (Method 1) for *Hyalocylis* (Pliocene), *Diacria*, *Cavolinia/Diacavolinia* (Miocene), and *Limacina* (Eocene). This was not the case for *Cuvierina* (Miocene) and *Creseis* (Eocene), for which crown ages appeared to be younger than the fossil evidence, derived at 4.42 and 12.12 mya, respectively. Derived crown group ages based on leaving out one fossil stem calibration per independent run (Method 2) are ~1–3 million years younger than ages derived in Method 1 for *Hyalocylis*, *Diacria*, *Cuvierina*, and *Creseis*, and ~8 million years younger for *Cavolinia-Diacavolinia* (Table 2). Derived ages from independent runs that left out one of the three IOP-calibrations each (Method 3) correspond best for *Hyalocylis striata* (derived at 2.99 mya), but are slightly older for *Clio pyramidata* (4.01 mya), and much older for the gymnosome *Cliopsis krohni* (8.62 mya). These ages correspond well with the ages of uncalibrated nodes in Methods 1 and 2 (Table 2).

**Table 2:** Overview of node ages as calibrated, as derived from independent runs without specific node calibrations, and as derived from two runs using all calibrations from a specific method.

Group	Calibration type	Calibrated/derived age	Age of crown group (95% confidence intervals; mya)			Calibration age (mya)
			Method 1	Method 2	Method 3	
Euthecosomata	Fossil: *Heliconoides* sp.	Calibrated	68.54 (76.1–65.9)	67.91 (73.2–65.8)	68.11 (74.4–65.8)	79–66
		*Derived*	*89*.*62 (116*.*3–66*.*4)*^3^	*75*.*79 (101*.*2–53*.*0)*^3^	*86*.*55 (116*.*6–57*.*4)*^3^	
Cavolinioidea^1^		*Derived*	*51*.*0 (60*.*0–41*.*75)*^4^		*42*.*5 (56*.*5–28*.*75)*^4^	
*Cavolinia—Diacavolinia*	Fossil: *Cavolinia zamboninii*	Calibrated	15.05 (17.6–14.0)	10.61 (14.1–6.5)		16–13.8
		*Derived*	*18*.*62 (29*.*1–10*.*2)*^3^	*10*.*45 (20*.*0–4*.*1)*^3^	*14*.*35 (23*.*0–7*.*2)*^4^	
*Clio pyramidata* (excl. f. *antarctica*)	IOP closure: Atlantic—Pacific	Calibrated			3.11 (4.9–1.5)	3.1
		*Derived*	*5*.*44 (13*.*0–0*.*9)*^4^	*3*.*72 (8*.*7–0*.*6)*^4^	*4*.*01 (9*.*9–0*.*9)*^3^	
*Clio convexa*/*pyramidata* group	None	*Derived*	*21*.*75 (34*.*5–9*.*75)*^4^	*14*.*5 (23*.*25–6*.*75)*^4^	*14*.*25 (25*.*75–6*.*75)*^4^	
*Clio cuspidata*/*recurva* group^2^	None	*Derived*	*3*.*0 (12*.*5–0*.*25)*^4^	*2*.*0 (8*.*5–0*.*25)*^4^	*2*.*0 (7*.*75–0*.*25)*^4^	
*Creseis*	Fossil: *Creseis corpulenta*	Calibrated	39.04 (41.0–38.2)	9.36 (21.9–2.3)		41–38
		*Derived*	*12*.*12 (26*.*2–3*.*0)*^3^	*9*.*09 (20*.*2–2*.*67)*^3^	*9*.*6 (22*.*0–2*.*9)*^4^	
*Cuvierina*	Fossil: *Cuvierina torpedo*	Calibrated	21.27 (23.1–20.6)	3.72 (10.0–0.7)		23–20.4
		*Derived*	*4*.*42 (11*.*2–0*.*8)*^3^	*3*.*37 (8*.*7–0*.*5)*^3^	*3*.*66 (9*.*2–0*.*8)*^4^	
*Diacria*	Fossil: *Diacria sangiorgii*	Calibrated	8.88 (12.0–7.5)	4.14 (11.0–0.7)		11.6–7.2
		*Derived*	*7*.*43 (20*.*6–1*.*1)*^3^	*5*.*19 (12*.*6–0*.*9)*^3^	*5*.*39 (15*.*0–0*.*9)*^4^	
*Hyalocylis*	Fossil: *Hyalocylis marginata*	Calibrated	3.19 (4.3–2.7)	2.25 (6.0–0.3)		3.6–2.6
		*Derived*	*3*.*82 (10*.*5–0*.*4)*^3^	*2*.*52 (6*.*5–0*.*3)*^3^	*See H*. *striata*.	
*Hyalocylis striata*	IOP closure: Atlantic—Pacific	Calibrated			2.85 (4.6–1.1)	3.1
		*Derived*	*See Hyalocylis*.	*See Hyalocylis*.	*2*.*99 (8*.*3–0*.*4)*^3^	
*Limacina*	Fossil: *Limacina gormani*	Calibrated	51.48 (58.1–48.3)			56–47.8
		*Derived*	*52*.*82 (66*.*5–35*.*2)*^3^	*50*.*33 (64*.*3–33*.*6)*^4^	*49*.*69 (64*.*2–32*.*7)*^4^	
Pseudothecosomata	None	*Derived*	*67*.*25 (98*.*0–36*.*5)*^4^	*56*.*75 (84*.*75–29*.*0)*^4^	*58*.*25 (86*.*0–30*.*25)*^4^	
Cymbuliidae	None	*Derived*	*29*.*0 (55*.*5–9*.*25)*^4^	*23*.*0 (46*.*0–7*.*0)*^4^	*23*.*75 (46*.*0–7*.*0)*^4^	
Gymnosomata^2^	None	*Derived*	*75*.*0 (103*.*75–47*.*0)*^4^	*63*.*25 (89*.*25–38*.*5)*^4^	*65*.*25 (92*.*0–40*.*25)*^4^	
*Cliopsis krohni*	IOP closure: Atlantic—Pacific	Calibrated			3.47 (5.2–1.8)	3.1
		*Derived*	*10*.*8 (26*.*0–1*.*8)*^4^	*7*.*69 (19*.*3–1*.*0)*^4^	*8*.*62 (21*.*2–0*.*8)*^3^	
Pteropods vs. outgroup taxa	Secondary: *Clione antarctica* vs. *Aplysia californica*	Calibrated	115.63 (142.8–90.1)	104.47 (130.7–80.6)	105.96 (132.1–82.0)	170–90
		*Derived*	*94*.*14 (134*.*4–68*.*3)*^3^	*74*.*95 (94*.*2–66*.*3)*^3^	*74*.*63 (90*.*3–66*.*5)*^3^	

The Gymnosomata and of the *Clio cuspidata*/*recurva* group are supported in time-calibrated, but not in unconstrained multi-gene Maximum Likelihood analyses. The monophyly of uncoiled Euthecosomata (Cavolinioidea) is not supported with a posterior probability of >95 in calibration Method 2 (*see* Table 1). mya = million years ago.

^1^ Not supported with posterior probability of >0.95 in molecular clock Method 2

^2^ Supported monophyly in molecular clock, but not in ML analyses

^3^ Derived from independent run without calibration of this node (100 million generations, 10% burn-in)

^4^ Derived from 2 runs using all calibrations from specific method (2 * 100 million generations, 10% burn-in each)

At the higher taxonomic levels (Pteropoda, Euthecosomata, Pseudothecosomata, Gymnosomata) node ages are more variable. The derived age of pteropods versus the outgroup taxa *Aplysia californica* and *Bulla striata* results in a younger age compared to the secondary calibration of this node: 94–74 mya instead of 116–104 mya with large 95% error ranges (Table 2). When not used as a fossil crown calibration, the derived age of the euthecosomes appears to be overestimated by all three calibration methods compared to its fossil age (90–75 mya instead of 79–66 mya), but all with large 95% error ranges of 50–59 million years. Derived ages of the pseudothecosomes are 67.25, 56.75, and 58.25 mya, and for the gymnosomes they are 75.0, 63.25, and 65.25 mya, based on calibration methods 1, 2, and 3, respectively (Fig 3; S7 and S8 Figs).

## Discussion

### Taxonomy of Pteropoda

Generally, our reconstructed molecular phylogenies match the current morphology-based taxonomy of pteropods. Most species and genera are well resolved and confirmed to be monophyletic and hence provide a useful framework for ecological and evolutionary studies of the group. A taxonomic division of pteropods into euthecosomes, pseudothecosomes, and gymnosomes is also consistent with our data, although their phylogenetic interrelationships remain unresolved. We cannot confirm that euthecosomes are more closely related to pseudothecosomes than they are to gymnosomes, as reflected in current taxonomy, in which the eu- and pseudothecosomes represent the order Thecosomata based on shared morphological characters that set them apart from the order Gymnosomata. The parapodia (wing-like structures) of thecosomes are positioned differently compared to gymnosomes with respect to the head, mouth, and foot-lobes [82,83]. Resolving higher-level taxonomic relationships in pteropods will require additional molecular evidence, such as could be provided by transcriptome data that enable a phylogenomic approach.

All our analyses recover the monophyly of uncoiled euthecosomes supporting the validity of the superfamily Cavolinioidea. We see no reason for changing this name to Orthoconcha, as suggested by [35]. However, the current taxonomy of families within the Cavolinioidea was not supported by our phylogenetic analyses. Currently valid families are Cavoliniidae (*Cavolinia*, *Diacavolinia*, *Diacria*), Cliidae (*Clio*), Creseidae (*Creseis*, *Hyalocylis*, *Styliola*), and Cuvierinidae (*Cuvierina*). This classification corresponds with the overall shape of shells: complex and round for Cavoliniidae, wide and conical for Cliidae, narrow and conical for Creseidae, and bottle-shaped for Cuvierinidae (Fig 2). We only found one supported subdivision within Cavolinioidea based on ML phylogenies, with *Creseis* as a monophyletic group and sister clade to the cluster of genera comprising *Cavolinia*, *Clio*, *Cuvierina*, *Diacavolinia*, *Diacria*, *Hyalocylis*, and *Styliola* (Fig 2). The time-calibrated phylogenies support additional subdivisions within Cavolinioidea (e.g. *Cavolinia* + *Diacavolinia* + *Hyalocylis*); however, they do not reflect current family-level taxonomy either.

The genus *Clio* was paraphyletic in the multi-gene phylogenies, with the well-resolved clade comprising *Clio convexa*, *C*. *pyramidata*, and *C*. *pyramidata antarctica*, being sister to another clade comprising *C*. *cuspidata* and *C*. *recurva* that was only supported in time-calibrated Bayesian phylogenies. Morphological characteristics of the two groups support this division: *Clio convexa*, *C*. *pyramidata*, and *C*. *pyramidata antarctica* have an elongated protoconch (larval shell) with a subtle transition into the apical spine (larval shell tip), whereas *Clio cuspidata* and *C*. *recurva* have a round protoconch with a sharp transition into the apical spine (e.g. [22]). More genetic information is required to clarify the phylogenetic position of *C*. *cuspidata* and *C*. *recurva*, and additional *Clio* taxa should be included in the analysis, such as the deep-dwelling *C*. *andreae*, *C*. *chaptalii*, *C*. *polita*, and *C*. *piatkowskii*.

*Diacavolinia*, introduced as a genus by [29] was previously included in *Cavolinia* as the single species *Cavolinia longirostris*. We recovered *Diacavolinia* as nested within *Cavolinia*. Specimens identified as *Diacavolinia* sp. 1 were from the Atlantic Ocean and match the Atlantic sequences from [39] and *Diacavolinia* Group 1 in [41]. Pacific individuals used in single-gene COI analyses (*Diacavolinia* sp. 2) match the Pacific sequences from [39] as well as *Diacavolinia* Group 12 in [41]. The high levels of morphological and genetic diversity found for both *Diacavolinia* and *Cavolinia* suggests that more taxon sampling across all oceans and additional genetic markers are needed to resolve their taxonomy.

The coiled euthecosomes or Limacinoidea consist of a single family, the Limacinidae, which appears to be paraphyletic with respect to the uncoiled euthecosomes. The genera *Heliconoides*, *Limacina*, and *Thielea* are genetically more divergent than the Cavolinioidea genera. This may be a result of their earlier origin compared to uncoiled euthecosomes, and/or higher evolutionary rates, as observed for the conservative markers 28S and 18S. [35] proposed to rename *Heliconoides inflatus* (listed in their study as *Limacina inflata*) to *Embolus inflata* to emphasize a putative soft polytomy between *Heliconoides inflatus* and other coiled euthecosomes, however, based on the available evidence, we consider the position of *Heliconoides* as unresolved. Moreover, earliest fossil occurrences do not support the presence of a hard polytomy of coiled euthecosome genera [10,22,74] (*see* next section). Finally, *Embolus* is currently considered a synonym of *Heliconoides* and represents an invalid junior homonym of *Embolus*, a genus of echinoderm [25].

The single and multi-gene trees strongly supported the taxonomy of the pseudothecosome families Peraclidae (*Peracle*) and Cymbuliidae (*Cymbulia*, *Corolla*, *Gleba*). However, pseudothecosome species remain poorly studied compared to euthecosomes, and additional taxon sampling of all genera, including *Desmopterus*, is required to resolve their taxonomy.

Only four out of six families and eight of 19 genera of Gymnosomata were included in our analyses, and thus phylogenetic relationships should be regarded as tentative. Our analyses did not support the current taxonomy of the gymnosome families examined: Clionidae (*Clione*, *Thliptodon*), Cliopsidae (*Cliopsis*), Notobranchaeidae (*Notobranchaea*), and Pneumodermatidae (*Pneumoderma*, *Pneumodermopsis*, *Schizobrachium*, *Spongiobranchaea*). We found *Clione* as the most likely sister taxon of *Notobranchaea*, and *Thliptodon* grouped separately from all other gymnosome taxa.

### Evolutionary history

Based on a molecular clock approach using fossils and the IOP as calibrations, we propose a scenario of pteropod evolution, in which this group originated during the Late Cretaceous, and most extant genera evolved during the Late Oligocene and Miocene. The euthecosomes, pseudothecosomes, and gymnosomes most likely evolved during the final stages of the Late Cretaceous (79–66 mya) from which the first *Heliconoides* euthecosome fossil is known [74]. Interestingly, this is earlier than previously thought as the time of divergence between the euthecosomes and pseudothecosomes was estimated at 58.6/57.3 mya by [35] based on two different molecular clock methods. However, this may be due to their incorrect assumption that the Early-Eocene (Ypresian) thecosome genus *Altaspiratella* belongs to the Pseudothecosomata.

Among the coiled euthecosomes, the evolution of *Heliconoides* in the Late Cretaceous was followed by the origin of *Limacina* in the Early Eocene at ~56 mya based on fossil records and our time-calibrated molecular phylogeny. The Paleocene-Eocene thermal maximum (PETM) at ~56 mya was probably a critical event in pteropod evolution. Although some studies have suggested that modern pteropod families appeared only after the PETM (e.g. [11]), a recent study of a PETM assemblage of pteropods demonstrated the presence of *Limacina* and *Heliconoides* as well as the extinct genus *Altaspiratella* in this period [10]. Based on fossil evidence, it was suggested that most genera that originated after the PETM went extinct before the end of the Eocene at 33.9 mya, when global ocean temperatures dropped [84,85].

We estimated that uncoiled euthecosomes evolved during the Early to Middle Eocene at 51–42 mya, probably from a coiled euthecosome ancestor, as was earlier suggested by [86] and [87]. Putative successive stages of despiralisation have been observed in some pteropod species from the Eocene (Ypresian, 56.0–47.8 mya; Lutetian, 47.8–41.3 mya), as in particular species of the limacinid genus *Altaspiratella*, leading to the creseid genera *Camptoceratops* and *Euchilotheca* [44]. *Camptoceratops priscus* was used by [35] to calibrate the origin of the euthecosomes, but based on its intermediate morphology between coiled and uncoiled taxa its phylogenetic position is considered uncertain. *Creseis* appeared as the first extant uncoiled genus at 41–38 mya (Middle Eocene: Bartonian), followed by a *Clio* representative at 38–34 mya (Late Eocene: Priabonian) [88,89].

During the Oligocene and Miocene, the (sub)tropical connectivity between the Atlantic and Indian oceans decreased and finally ceased as a result of closure of the Miocene Tethys Sea (Terminal Tethyan Event TTE) [50,51], and during this period of oceanographic and climatic change many extant pteropod genera appeared. The uncoiled euthecosome genera *Cavolinia*, *Cuvierina*, *Diacria*, *Styliola*, and the *Clio convexa*/*pyramidata* group originated during the late Oligocene and Miocene between 40 and 15 mya. [35] estimated that the family Cavoliniidae evolved 47.1/30.0 mya, but we did not find evidence for its monophyly. Most pseudothecosome and gymnosome genera probably originated during the Late Oligocene and Miocene [90]. We could not estimate the age of the bathypelagic, coiled euthecosome *Thielea*, but rare fossil evidence suggests at least a Miocene (Tortonian, 11.6–7.2 mya) origin [22].

The uncoiled euthecosomes *Hyalocylis*, *Diacavolinia* and the *Clio cuspidata*/*recurva* group appear to be of more recent, Pliocene origin, when the Isthmus of Panama (IOP) became totally emergent, and fossil evidence supports this age (5.3–2.6 mya) [22,43]. Hence, the age of *Hyalocylis* appears to have been overestimated by [35] at 38.5/16.1 mya. This age rather corresponds with the Late Eocene origin of *Praehyalocylis*, which is the supposed ancestor of *Hyalocylis* [90]. The IOP emergence led to a further reduction in (sub)tropical connectivity between the Atlantic and Indo-Pacific oceans. Isolation of (sub)tropical Atlantic and Pacific populations probably did not occur at the same time for all species. For example, the gymnosome *Cliopsis krohni* may require deeper maximum depths than *Hyalocylis striata* and *Clio pyramidata*, occurring as deep as 1500m [91]. This could explain the earlier divergence between Atlantic and Pacific populations of *Cliopsis krohni* compared to *Hyalocylis striata* and *Clio pyramidata*. Recent molecular studies of the (sub)tropical *Cuvierina* and *Diacavolinia* taxa also suggested isolation of Atlantic and Indo-Pacific taxa [39–41].

### Pteropods in the Anthropocene

We present a framework for understanding the evolutionary relationships among pteropods with a focus on shelled euthecosome species based on an integrated analysis of three molecular markers, fossil evidence, and vicariance events. Although not all phylogenetic relationships could be resolved, this study provides new data on the diversity of pteropods, which is an essential first step for their use as bio-indicators of the ongoing effects of ocean acidification (e.g. [6]). Recent advances in high-throughput sequencing will allow sampling across the entire genome and will result in better resolved and more robust phylogenies, especially at higher taxonomic levels. Anthropogenic carbon input into the atmosphere and the ocean is occurring over the course of just hundreds instead of thousands of years. Hence, the impacts on surface ocean pH are more precipitous now than during the PETM [8,9]. Although the PETM may be the most comparable ocean acidification event in the past, its preceding climate conditions were very different from the present day. Continents were configured differently and the global climate was warmer [9,92]. [93] found that the current sensitivity of marine ecosystems to carbon perturbation is likely higher than during the PETM because the ocean’s chemistry might have differed substantially. All in all, it is likely that the effects of ocean acidification on pteropods will be unprecedented, and this key group of the zooplankton community will be among the most severely affected.

## Supporting information

S1 Fig Maximum Likelihood phylogeny of pteropods based on Cytochrome Oxidase I sequences (N = 117 sequences of 656 basepairs). Black squares represent a bootstrap support of ≥80%, with small, medium and large black squares representing support within genera, of genera, and above genus level, respectively. Abbreviations ATL, PAC, and IND denote Atlantic, Pacific, and Indian Ocean origins, respectively, including their sectors in the Southern Ocean; ARC denotes the Arctic Sea.
(PDF)

S2 Fig Maximum Likelihood phylogeny of pteropods based on Cytochrome Oxidase I protein sequences (N = 117 sequences of 218 amino acids). Black squares represent a bootstrap support of ≥80%, with small, medium and large black squares representing support within genera, of genera, and above genus level, respectively. Abbreviations ATL, PAC, and IND denote Atlantic, Pacific, and Indian Ocean origins, respectively, including their sectors in the Southern Ocean; ARC denotes the Arctic Sea.
(PDF)

S3 Fig Maximum Likelihood phylogeny of pteropods based on 28S sequences (N = 87 sequences of 941 basepairs). Black squares represent a bootstrap support of ≥80%, with small, medium and large black squares representing support within genera, of genera, and above genus level, respectively. Abbreviations ATL, PAC, and IND denote Atlantic, Pacific, and Indian Ocean origins, respectively, including their sectors in the Southern Ocean.
(PDF)

S4 Fig Maximum Likelihood phylogeny of pteropods based on 18S sequences (N = 52 sequences of 1683 basepairs). Black squares represent a bootstrap support of ≥80%, with small, medium and large black squares representing support within genera, of genera, and above genus level, respectively. Abbreviations ATL, PAC, and IND denote Atlantic, Pacific, and Indian Ocean origins, respectively, including their sectors in the Southern Ocean.
(PDF)

S5 Fig Combined Maximum Likelihood tree based on Cytochrome Oxidase I protein sequences and 28S and 18S DNA sequences including long-branch taxa (78 sequences, max. two sequences per taxon per ocean). Black squares represent bootstrap support ≥80%, with small, medium and large black squares representing support within genera, of genera, and above genus level, respectively. Abbreviations ATL, PAC, and IND denote Atlantic, Pacific, and Indian Ocean origins, respectively, including their sectors in the Southern Ocean.
(PDF)

S6 Fig Combined Maximum Likelihood phylogeny using the same dataset as for the molecular clock analyses (46 sequences, max. one sequence per taxon per ocean). Black squares represent bootstrap support ≥80%, with small, medium and large black squares representing support within genera, of genera, and above genus level, respectively. Abbreviations ATL, PAC, and IND denote Atlantic, Pacific, and Indian Ocean origins, respectively, including their sectors in the Southern Ocean.
(PDF)

S7 Fig Pteropod phylogeny based on a fossil-calibrated molecular clock approach (46 sequences, max. one sequence per taxon per ocean) following Method 2 with stem calibrations based on the oldest known fossils of *Hyalocylis*, *Diacria*, *Cavolinia*, *Cuvierinia*, *Creseis*, and euthecosomes (*see* Table 1). Calibrations are indicated with stars. Error bars (95%) are shown only for clades with posterior probabilities ≥0.95 in green for uncoiled euthecosomes, purple for coiled euthecosomes, red for pseudothecosomes, and blue for gymnosomes. Abbreviations ATL, PAC, and IND denote Atlantic, Pacific, and Indian Ocean origins, respectively, including their sectors in the Southern Ocean.
(PDF)

S8 Fig Time-calibrated molecular phylogeny of pteropods (46 sequences, max. one sequence per taxon per ocean) following Method 3 with crown calibrations of *Clio pyramidata*, *Hyalocylis striata*, and *Cliopsis krohni* based on the formation of the Isthmus of Panama (IOP) and the oldest known euthecosome fossil (*see* Table 1). Calibrations are indicated with stars. Error bars (95%) are shown only for clades with posterior probabilities ≥0.95 in green for uncoiled euthecosomes, purple for coiled euthecosomes, red for pseudothecosomes, and blue for gymnosomes. Abbreviations ATL, PAC, and IND denote Atlantic, Pacific, and Indian Ocean origins, respectively, including their sectors in the Southern Ocean.
(PDF)

S1 Table Overview of sequences used in combined and/or as single-gene phylogenetic analyses based on Cytochrome Oxidase I, 28S rRNA, and 18S rRNA. Numbers in the 9^th^ column indicate their use in (1) single-gene Maximum Likelihood (ML), combined ML and combined Bayesian phylogenies, (2) single-gene ML and combined ML phylogenies, or (3) single-gene ML phylogenies. An asterisk indicates long-branch taxa that were excluded in separate multi-gene ML phylogenetic analyses. Bp = number of basepairs per sequence. *Clione limacina antarctica* sequences were obtained from a transcriptome (T). Picture numbers indicated with an asterisk represent juvenile specimens (available in the Dryad digital repository [57]).
(PDF)
