# Supplementary figures and images for: Time-calibrated molecular phylogeny of pteropods
Source: PLoS One. 2017 Jun 12;12(6):e0177325. doi: 10.1371/journal.pone.0177325 (PMC5467808; doi:10.1371/journal.pone.0177325)

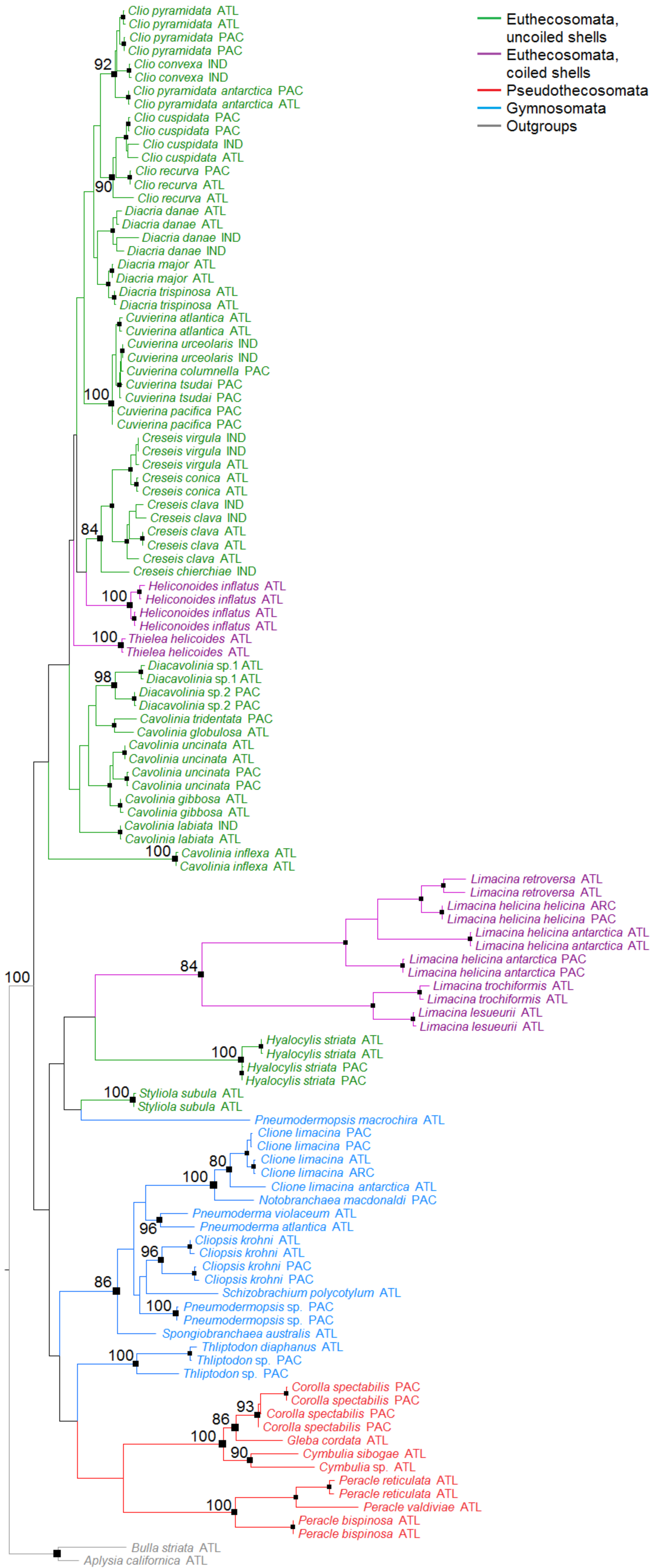

Supplement: S1 Fig — Black squares represent a bootstrap support of ≥80%, with small, medium and large black squares representing support within genera, of genera, and above genus level, respectively. Abbreviations ATL, PAC, and IND denote Atlantic, Pacific, and Indian Ocean origins, respectively, including their sectors in the Southern Ocean; ARC denotes the Arctic Sea. (PDF) [file pone.0177325.s001.pdf]

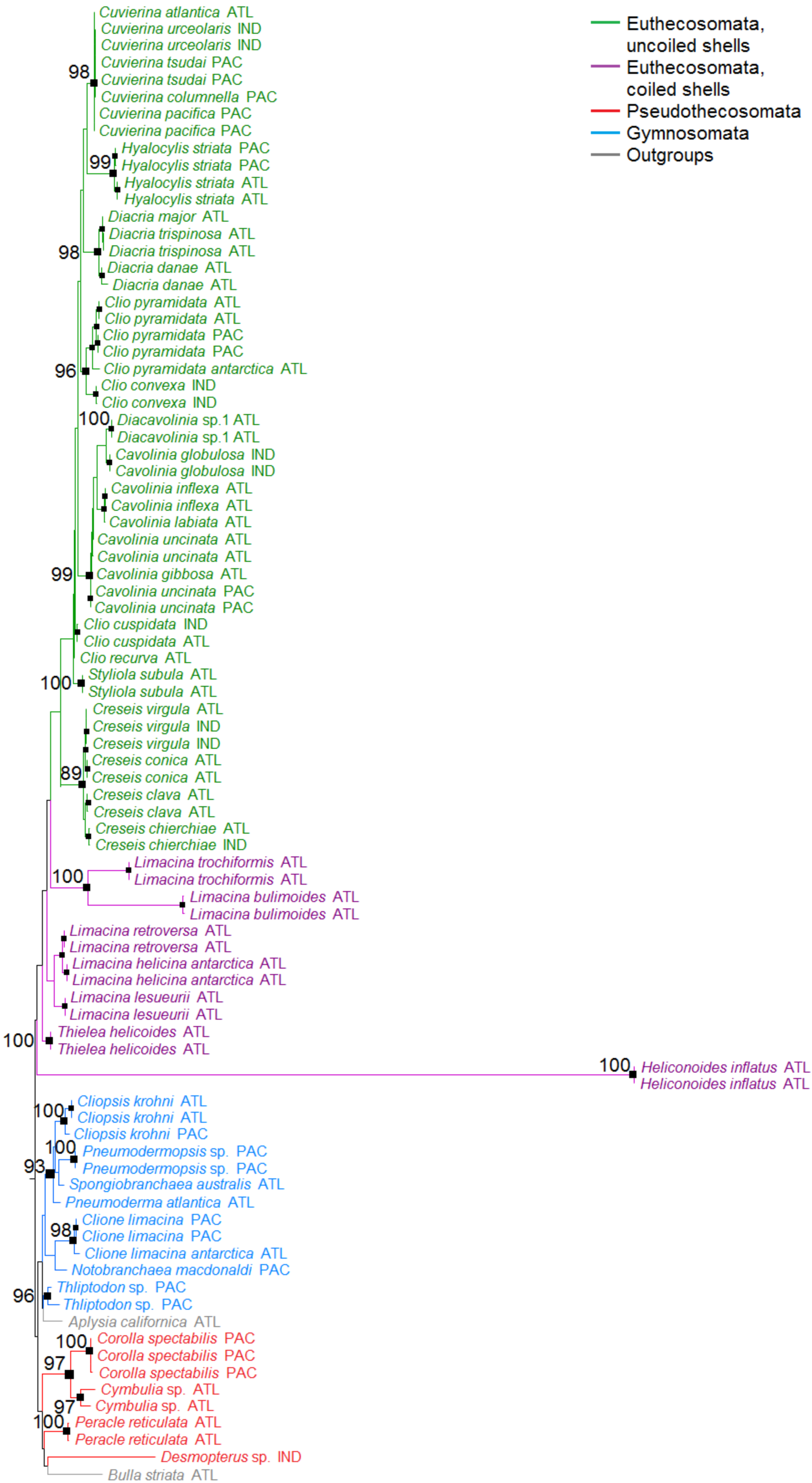

Supplement: S3 Fig — Black squares represent a bootstrap support of ≥80%, with small, medium and large black squares representing support within genera, of genera, and above genus level, respectively. Abbreviations ATL, PAC, and IND denote Atlantic, Pacific, and Indian Ocean origins, respectively, including their sectors in the Southern Ocean. (PDF) [file pone.0177325.s003.pdf]

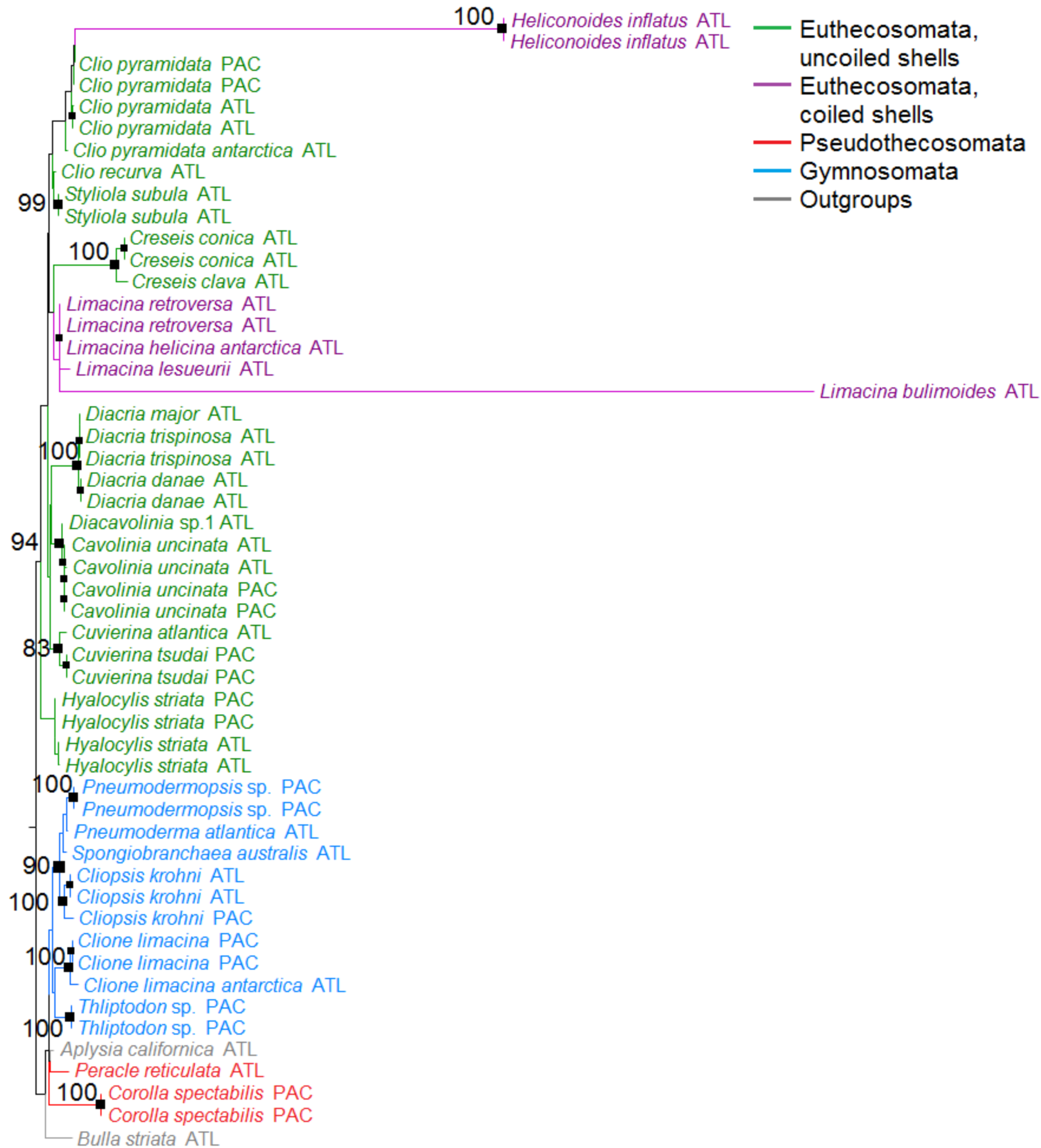

Supplement: S4 Fig — Black squares represent a bootstrap support of ≥80%, with small, medium and large black squares representing support within genera, of genera, and above genus level, respectively. Abbreviations ATL, PAC, and IND denote Atlantic, Pacific, and Indian Ocean origins, respectively, including their sectors in the Southern Ocean. (PDF) [file pone.0177325.s004.pdf]

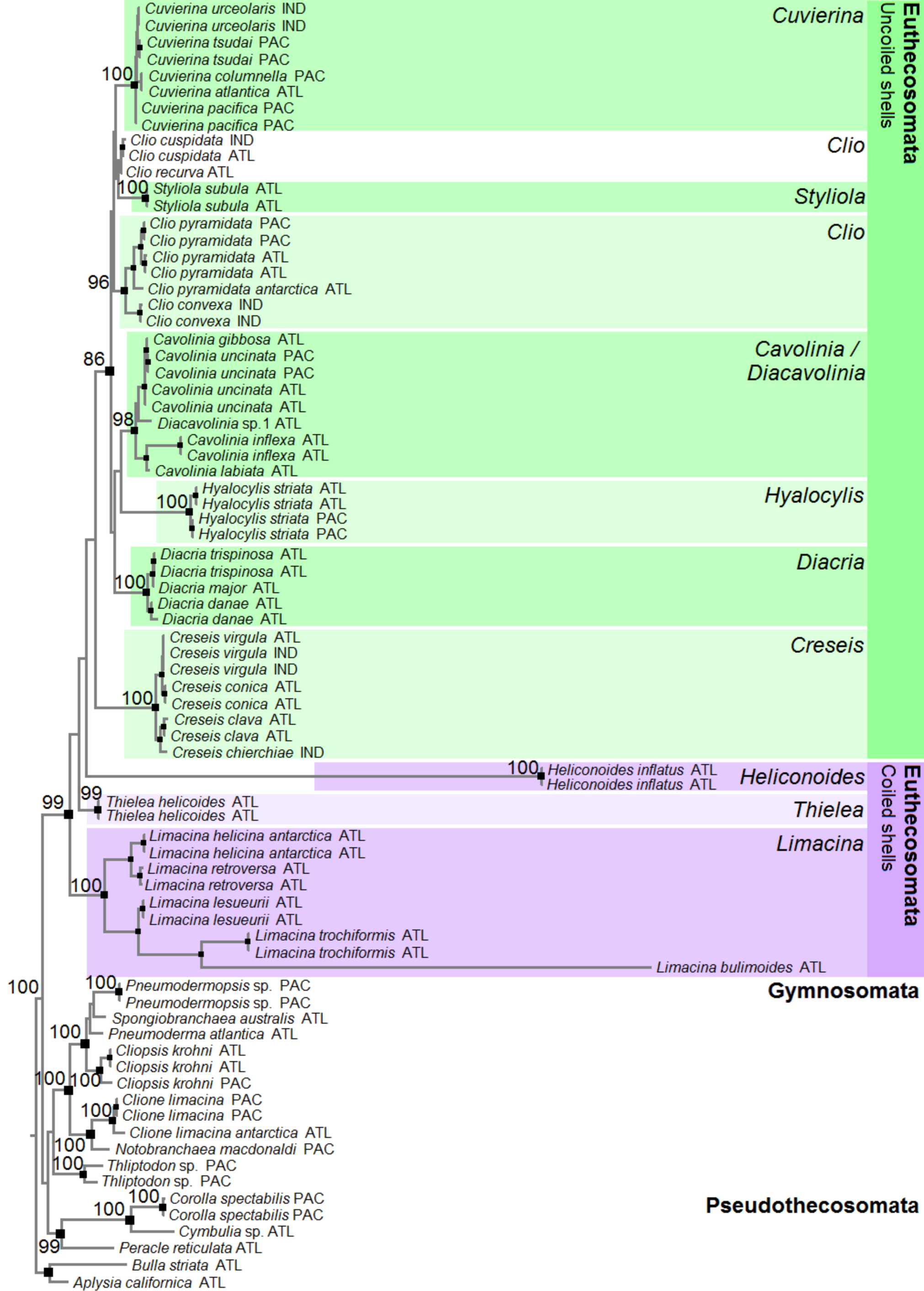

Supplement: S5 Fig — Black squares represent bootstrap support ≥80%, with small, medium and large black squares representing support within genera, of genera, and above genus level, respectively. Abbreviations ATL, PAC, and IND denote Atlantic, Pacific, and Indian Ocean origins, respectively, including their sectors in the Southern Ocean. (PDF) [file pone.0177325.s005.pdf]

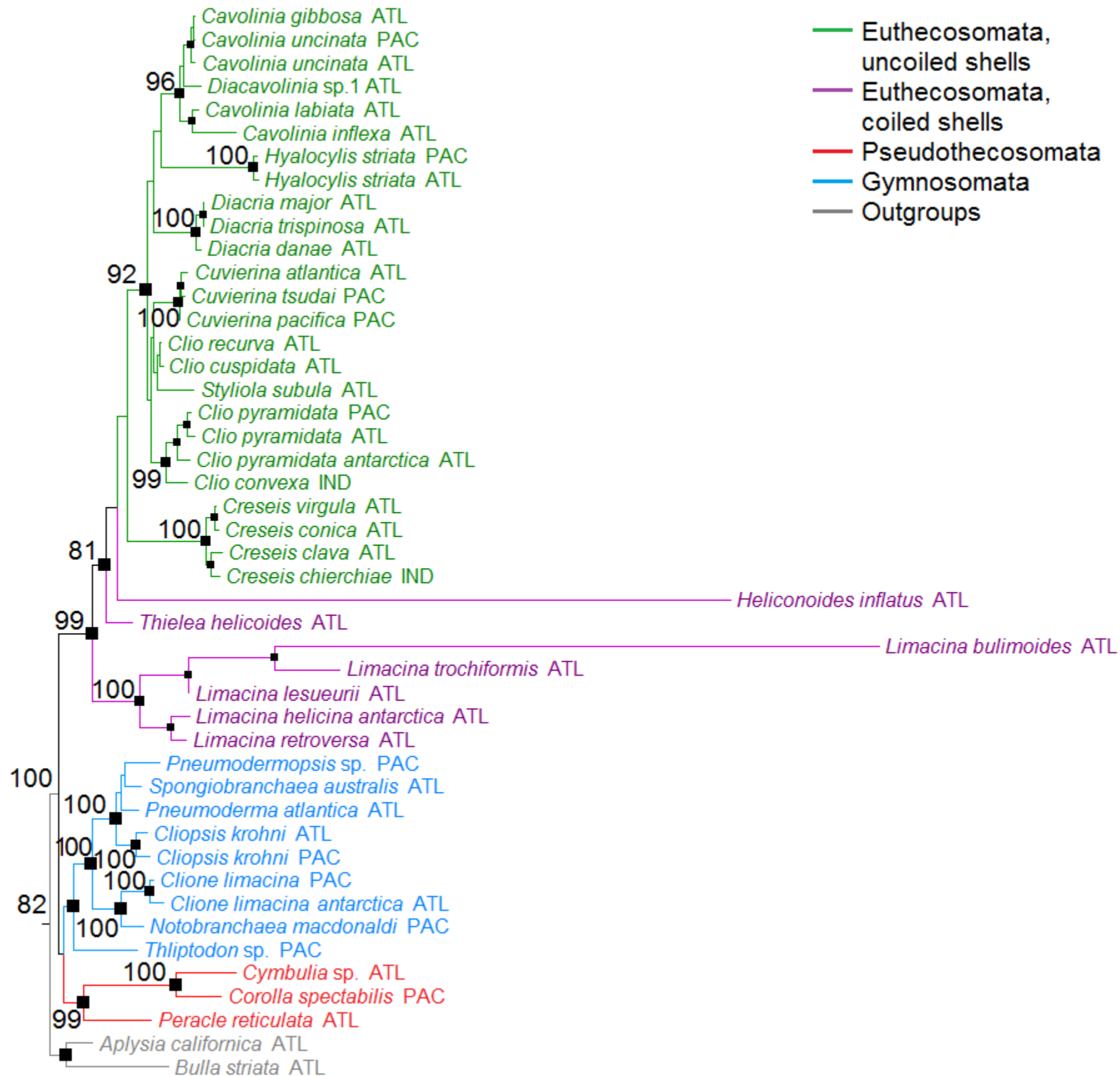

Supplement: S6 Fig — Black squares represent bootstrap support ≥80%, with small, medium and large black squares representing support within genera, of genera, and above genus level, respectively. Abbreviations ATL, PAC, and IND denote Atlantic, Pacific, and Indian Ocean origins, respectively, including their sectors in the Southern Ocean. (PDF) [file pone.0177325.s006.pdf]

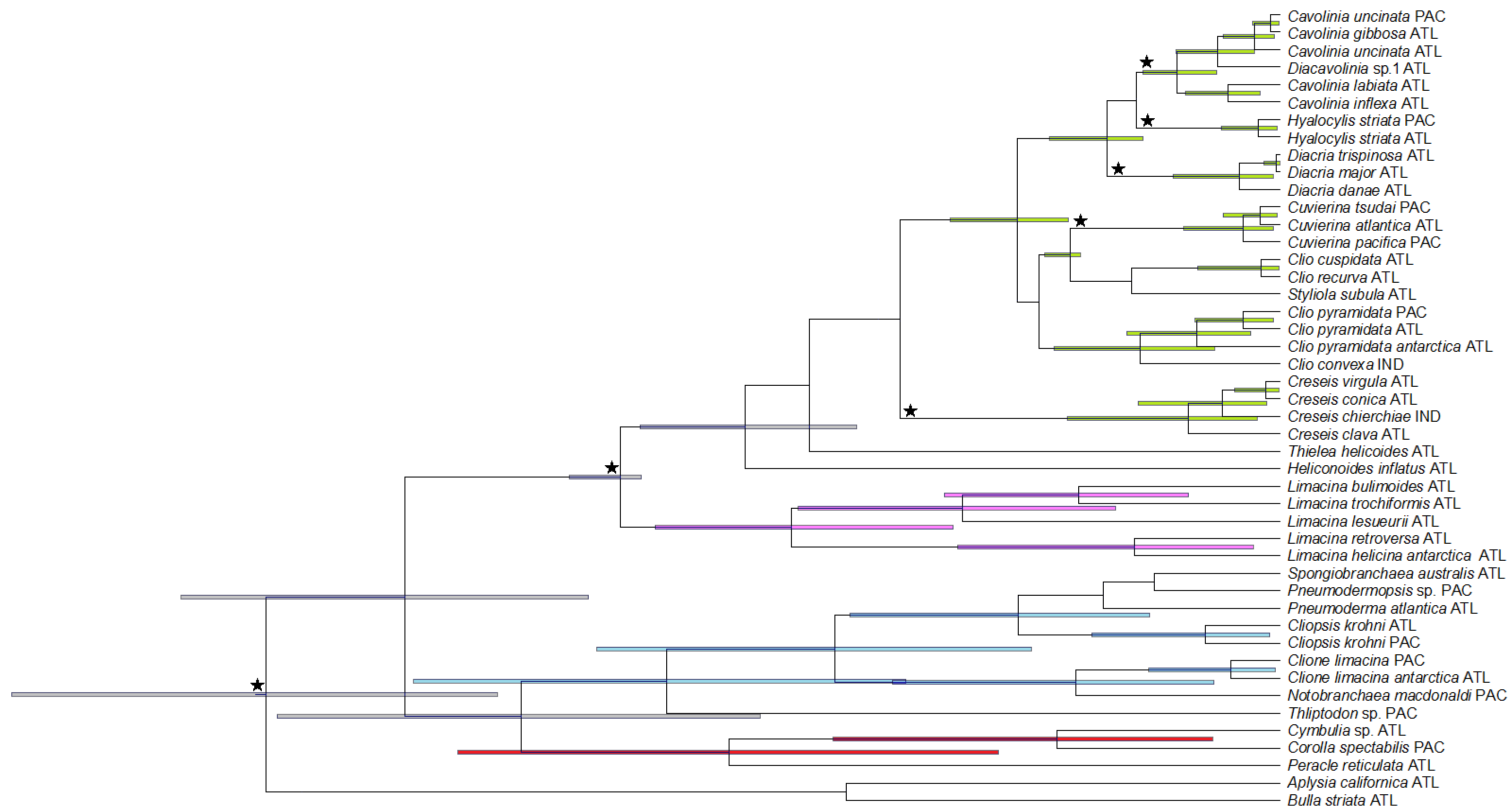

Supplement: S7 Fig — Calibrations are indicated with stars. Error bars (95%) are shown only for clades with posterior probabilities ≥0.95 in green for uncoiled euthecosomes, purple for coiled euthecosomes, red for pseudothecosomes, and blue for gymnosomes. Abbreviations ATL, PAC, and IND denote Atlantic, Pacific, and Indian Ocean origins, respectively, including their sectors in the Southern Ocean. (PDF) [file pone.0177325.s007.pdf]

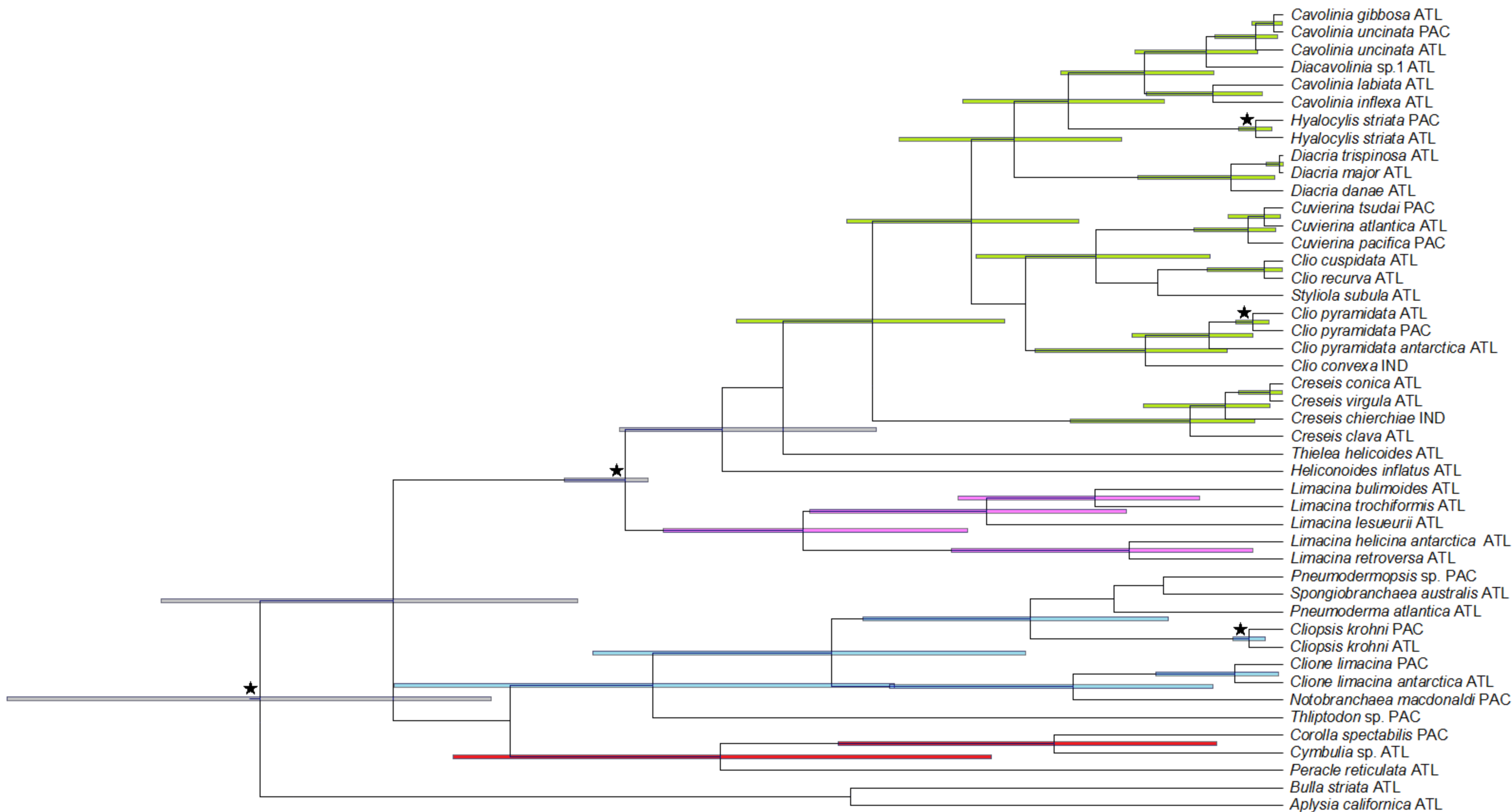

Supplement: S8 Fig — Calibrations are indicated with stars. Error bars (95%) are shown only for clades with posterior probabilities ≥0.95 in green for uncoiled euthecosomes, purple for coiled euthecosomes, red for pseudothecosomes, and blue for gymnosomes. Abbreviations ATL, PAC, and IND denote Atlantic, Pacific, and Indian Ocean origins, respectively, including their sectors in the Southern Ocean. (PDF) [file pone.0177325.s008.pdf]
