# Supplementary material for: Time-calibrated molecular phylogeny of pteropods
Source: PLoS One. 2017 Jun 12;12(6):e0177325. doi: 10.1371/journal.pone.0177325 (PMC5467808; doi:10.1371/journal.pone.0177325)
Supplement: S2 Fig — Black squares represent a bootstrap support of ≥80%, with small, medium and large black squares representing support within genera, of genera, and above genus level, respectively. Abbreviations ATL, PAC, and IND denote Atlantic, Pacific, and Indian Ocean origins, respectively, including their sectors in the Southern Ocean; ARC denotes the Arctic Sea. (PDF) [file pone.0177325.s002.pdf]

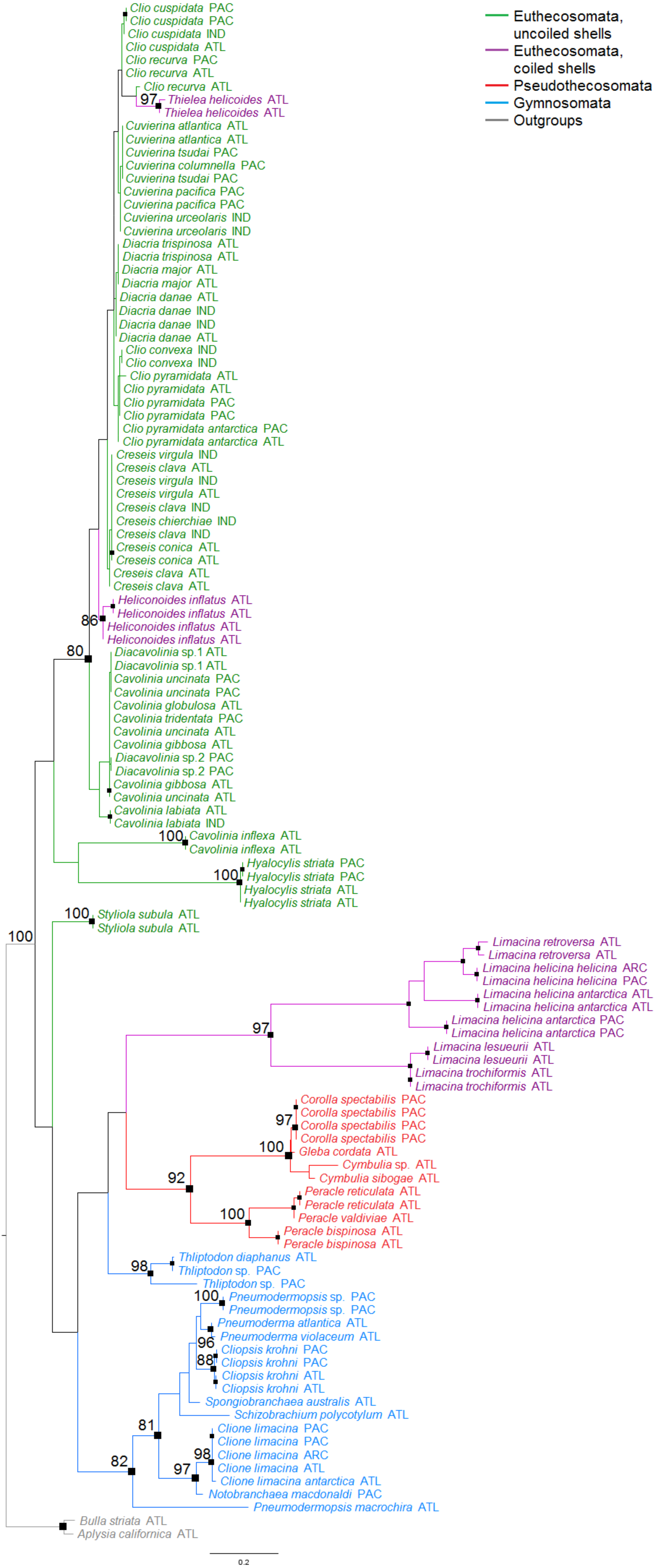

Euthecosomata, uncoiled shells
Euthecosomata, coiled shells
Pseudothecosomata
Gymnosomata
Outgroups
Clio cuspidata PAC
Clio cuspidata PAC
Clio cuspidata IND
Clio cuspidata ATL
Clio recurva PAC
Clio recurva ATL
Clio recurva ATL
97
Thielea helicoides ATL
Thielea helicoides ATL
Cuvierina atlantica ATL
Cuvierina atlantica ATL
Cuvierina tsudai PAC
Cuvierina columnella PAC
Cuvierina tsudai PAC
Cuvierina pacifica PAC
Cuvierina pacifica PAC
Cuvierina urceolaris IND
Cuvierina urceolaris IND
Diacria trispinosa ATL
Diacria trispinosa ATL
Diacria major ATL
Diacria major ATL
Diacria danae ATL
Diacria danae IND
Diacria danae IND
Diacria danae ATL
Clio convexa IND
Clio convexa IND
Clio pyramidata ATL
Clio pyramidata ATL
Clio pyramidata PAC
Clio pyramidata PAC
Clio pyramidata antarctica PAC
Clio pyramidata antarctica ATL
Creseis virgula IND
Creseis clava ATL
Creseis virgula IND
Creseis virgula ATL
Creseis clava IND
Creseis chierchiae IND
Creseis clava IND
Creseis conica ATL
Creseis conica ATL
Creseis clava ATL
Creseis clava ATL
Heliconoides inflatus ATL
Heliconoides inflatus ATL
86
Heliconoides inflatus ATL
Heliconoides inflatus ATL
80
Diacavolinia sp.1 ATL
Diacavolinia sp.1 ATL
Cavolinia uncinata PAC
Cavolinia uncinata PAC
Cavolinia globulosa ATL
Cavolinia tridentata PAC
Cavolinia uncinata ATL
Cavolinia gibbosa ATL
Diacavolinia sp.2 PAC
Diacavolinia sp.2 PAC
Cavolinia gibbosa ATL
Cavolinia uncinata ATL
Cavolinia labiata ATL
Cavolinia labiata IND
100
Cavolinia inflexa ATL
Cavolinia inflexa ATL
Hyalocylis striata PAC
100
Hyalocylis striata PAC
Hyalocylis striata ATL
Hyalocylis striata ATL
100
Styliola subula ATL
Styliola subula ATL
100
Limacina retroversa ATL
Limacina retroversa ATL
Limacina helicina helicina ARC
Limacina helicina helicina PAC
Limacina helicina antarctica ATL
Limacina helicina antarctica ATL
97
Limacina helicina antarctica PAC
Limacina helicina antarctica PAC
Limacina lesueurii ATL
Limacina lesueurii ATL
Limacina trochiformis ATL
Limacina trochiformis ATL
Corolla spectabilis PAC
Corolla spectabilis PAC
97
Corolla spectabilis PAC
Corolla spectabilis PAC
100
Gleba cordata ATL
Cymbulia sp. ATL
92
Cymbulia sibogae ATL
Peracle reticulata ATL
Peracle reticulata ATL
100
Peracle valdiviae ATL
Peracle bispinosa ATL
Peracle bispinosa ATL
Thliptodon diaphanus ATL
98
Thliptodon sp. PAC
Thliptodon sp. PAC
100
Pneumodermopsis sp. PAC
Pneumodermopsis sp. PAC
Pneumoderma atlantica ATL
Pneumoderma violaceum ATL
96
Cliopsis krohni PAC
88
Cliopsis krohni PAC
Cliopsis krohni ATL
Cliopsis krohni ATL
Spongiobranchaea australis ATL
Schizobrachium polycotylum ATL
81
Clione limacina PAC
Clione limacina PAC
98
Clione limacina ARC
82
97
Clione limacina ATL
Clione limacina antarctica ATL
Notobranchaea macdonaldi PAC
Pneumodermopsis macrochira ATL
Bulla striata ATL
Aplysia californica ATL
0.2
