# Supplementary material for: Time-calibrated molecular phylogeny of pteropods
Source: PLoS One. 2017 Jun 12;12(6):e0177325. doi: 10.1371/journal.pone.0177325 (PMC5467808; doi:10.1371/journal.pone.0177325)
Supplement: S1 Table — Numbers in the 9th column indicate their use in (1) single-gene Maximum Likelihood (ML), combined ML and combined Bayesian phylogenies, (2) single-gene ML and combined ML phylogenies, or (3) single-gene ML phylogenies. An asterisk indicates long-branch taxa that were excluded in separate multi-gene ML phylogenetic analyses. Bp = number of basepairs per sequence. Clione limacina antarctica sequences were obtained from a transcriptome (T). Picture numbers indicated with an asterisk represent juvenile specimens (available in the Dryad digital repository [57]). (PDF) [file pone.0177325.s009.pdf]

S1 Table. Overview of sequences used in combined and/or as single-gene phylogenetic analyses based on Cytochrome Oxidase I, 28S rRNA, and 18S rRNA. Numbers in the 9th column indicate their use in (1) single-gene Maximum Likelihood (ML), combined ML and combined Bayesian phylogenies, (2) single-gene ML and combined ML phylogenies, or (3) single-gene ML phylogenies. An asterisk indicates long-branch taxa that were excluded in separate multi-gene ML phylogenetic analyses. Bp = number of basepairs per sequence. *Clione limacina antarctica* sequences were obtained from a transcriptome (T). Picture numbers indicated with an asterisk represent juvenile specimens (available in the Dryad digital repository [57]).

Taxonomy & Species	Sample information							Genetics				Pictures
	Reference	Collection date	Latitude	Longitude	Cruise	Station	Ocean/sea/region	Used for	CO1	28S	18S	N
**Thecosomata, Euthecosomata**												
**Cavolinoidea**												
Cavolinia gibbosa	[35]	-	-	-	ECOSUR	-	Caribbean Sea	1	KC774033	KC774104		
Cavolinia gibbosa	[36]	2006-04-13	33°31'N	69°58'W	RHB0603	-	North Atlantic Ocean	3	FJ876856			
Cavolinia globulosa	[35]	-	-	-	TARA	41	Gulf of Aden	3		KC774101		
Cavolinia globulosa	[35]	-	-	-	TARA	42	Indian Ocean (Maldives)	3		KC774141		
Cavolinia globulosa	[36]	2006-04-25	14°00'N	55°00'W	RHB0603	-	North Atlantic Ocean	3	FJ876857			
Cavolinia inflexa	New	2008-10-20	11°49'N	32°49'W	AMT18	44	North Atlantic Ocean	2	MF048913	MF048966		2
Cavolinia inflexa	New	2008-10-20	11°49'N	32°49'W	AMT18	44	North Atlantic Ocean	1	MF048914	MF048967		2
Cavolinia labiata	[35]	-	-	-	TARA	52	Indian Ocean (East of Madagascar)	3	KC774038			
Cavolinia labiata	[35]	-	-	-	TARA	66	South Atlantic Ocean (Cape Town)	1	KC774037	KC774099		
Cavolinia tridentata atlantica	[36]	2007-01-22	46°21'S	140°32'E	Aurora_Australis_2007.3	-	Southern Ocean (South of Australia)	3	FJ876861			
Cavolinia uncinata	New	2011-01-31	18°07'N	87°44'W	GU1101	91	Caribbean Sea	1	MF048915	MF048968	MF049019	9
Cavolinia uncinata	New	2012-09-04	39°55'N	67°27'W	NOAA PC201205	ST23P4	Northwest Atlantic Ocean	2	MF048916	MF048969	MF049020	1
Cavolinia uncinata	New	2012-02-24	24°22'N	109°13'W	MBARI GOC 2012	D342BW	Gulf of California	1	MF048917	MF048970	MF049021	1
Cavolinia uncinata	New	2012-02-24	24°22'N	109°13'W	MBARI GOC 2012	D342BW	Gulf of California	2	MF048918	MF048971	MF049022	
Clio convexa	[35]	-	-	-	TARA	42	Indian Ocean (Maldives)	1	KC774063	KC774105		
Clio convexa	[35]	-	-	-	TARA	53	Indian Ocean (East of Madagascar)	2	KC774069	KC774093		
Clio cuspidata	[40]	2008-10-27	12°50'S	25°00'W	AMT18	66	South Atlantic Ocean	1	KP292789	KP292650		2
Clio cuspidata	[35]	-	-	-	TARA	42	Indian Ocean (Maldives)	2	KC774064	KC774098		
Clio cuspidata	[36]	2007-02-08	50°00'S	149°25'E	Aurora_Australis_2007.3	-	Southern Ocean (South of Australia)	3	FJ876869			
Clio cuspidata	[36]	2007-02-08	50°00'S	149°25'E	Aurora_Australis_2007.3	-	Southern Ocean (South of Australia)	3	FJ876870			
Clio pyramidata antarctica	New	2008-11-07	45°13'S	44°37'W	AMT18	101	South Atlantic / Southern Ocean	1	MF048919	MF048972	MF049023	1
Clio pyramidata antarctica	[36]	2007-02-08	50°00'S	149°25'E	Aurora_Australis_2007.3	-	Southern Ocean (South of Australia)	3	FJ876876			
Clio pyramidata lanceolata	New	2000-03	43°30'N	07°12'E	KP	-	Mediterranean Sea (Ligurian Sea: Villefranche-Sur-Mer)	2	MF048920	MF048973	MF049024	
Clio pyramidata pyramidata	[40]+New	2008-10-15	24°45'N	40°05'W	AMT18	27	North Atlantic Ocean	1	KP292791	KP292652	MF049025	2
Clio pyramidata	New	2012-02-17	24°11'N	109°38'W	MBARI GOC 2012	D333T	Gulf of California	1	MF048921	MF048974	MF049026	
Clio pyramidata	New	2012-02-17	24°11'N	109°38'W	MBARI GOC 2012	D333T	Gulf of California	2	MF048922	MF048975	MF049027	
Clio recurva	[40]+New	2004-06-09	59°58'N	25°45'W	MAR-ECO	2	North Atlantic Ocean	1	KP292790	KP292651	MF049028	
Clio recurva	New	2004-06-19	51°34'N	33°17'W	MAR-ECO	16	North Atlantic Ocean	3	MF048923			1
Clio recurva	[36]	2007-01-20	45°00'S	142°59'E	Aurora_Australis_2007.3	-	Southern Ocean (South of Australia)	3	FJ876880			

Creseis chierchiae	[35]	-	-	-	CRER 2	-	Caribbean Sea (Yucatan/Belize)	3		KC774136		
Creseis chierchiae	[35]	-	-	-	TARA	41	Gulf of Aden	1	KC774044	KC774137		
Creseis clava	New	2011-01-25	18°43'N	87°40'W	GU1101	53	Caribbean Sea	1			MF049029	5
Creseis clava	[38]	2007-01-21	18°47'N	87°08'W	GU0701	35	Caribbean Sea	3	HM385035			1
Creseis clava	[35]	-	-	-	TARA	41	Gulf of Aden	3	KC774052			
Creseis clava	[35]	-	-	-	CRER 2	-	Caribbean Sea (Yucatan/Belize)	1	KC774053	KC774125		
Creseis clava	[35]	-	-	-	ANTEDON	-	Mediterranean Sea (Gulf of Lyon, Cassidaigne)	2	KC774054	KC774126		
Creseis clava	[36]	2006-11-17	16°01'S	119°19'E	Galathea_2006	-	Indian Ocean	3	FJ876888			
Creseis conica	New	2011-01-31	18°07'N	87°44'W	GU1101	91	Caribbean Sea	1		MF048976	MF049030	2
Creseis conica	New	2011-01-31	18°07'N	87°44'W	GU1101	91	Caribbean Sea	1	MF048924	MF048977		2
Creseis conica	New	2011-01-31	18°07'N	87°44'W	GU1101	91	Caribbean Sea	2	MF048925		MF049031	2
Creseis virgula	[35]	-	-	-	ECOSUR	-	Caribbean Sea (Yucatan/Belize)	1	KC774045	KC774128		
Creseis virgula	[35]	-	-	-	TARA	34	Red Sea	2	KC774046	KC774129		
Creseis virgula	[35]	-	-	-	TARA	42	Indian Ocean (Maldives)	2	KC774047	KC774130		
Cuvierina atlantica	[40]+New	2004-07-01	41°29'N	28°19'W	MAR-ECO	36	North Atlantic Ocean	1	KP292669		MF049032	2
Cuvierina atlantica	[40]	2004-07-01	41°29'N	28°19'W	MAR-ECO	36	North Atlantic Ocean	1	KP292675	KP292622		2
Cuvierina columnella	[40]	2001-12-24	36°05'S	149°29'W	DRFT07RR	11	South Pacific Ocean	2	KP292728	KP292634		2
Cuvierina tsudai	[40]+New	2010-09-29	27°08'N	125°33'E	R/V Tansei-Maru KT-10-20	5	Northwest Pacific Ocean (East China Sea)	2		KP292642	MF049033	2
Cuvierina tsudai	[40]+New	2010-09-29	27°08'N	125°33'E	R/V Tansei-Maru KT-10-20	5	Northwest Pacific Ocean (East China Sea)	1	KP292767	MF048978	MF049034	2
Cuvierina tsudai	[40]	2010-09-29	27°08'N	125°33'E	R/V Tansei-Maru KT-10-20	5	Northwest Pacific Ocean (East China Sea)	2	KP292768			2
Cuvierina pacifica	[40]	2012-01-18	23°00'S	100°00'W	KH-11-10	21	South Pacific Ocean	1	KP292780	KP292647		2
Cuvierina pacifica	[40]	2001-10-14	29°05'S	176°09'W	COOK14MV	19	South Pacific Ocean	2	KP292785	KP292648		2
Cuvierina urceolaris	[40]	2003-06-06	12°52'S	94°26'E	VANC10MV	22	Indian Ocean	2	KP292729	KP292635		2
Cuvierina urceolaris	[35]	-	-	-	TARA	52	Indian Ocean (East of Madagascar)	2	KC774071	KC774107		
Diacavolinia longirostris (sp. 1)	[35]	-	-	-	ECOSUR	-	Caribbean Sea (Yucatan/Belize)	3		KC774118		
Diacavolinia sp. 1	New	2008-10-22	5°20'N	28°31'W	AMT18	50	North Atlantic Ocean	1	MF048926	MF048979	MF049035	
Diacavolinia sp. 1	New	2008-10-23	2°47'N	26°51'W	AMT18	56	North Atlantic Ocean	3	MF048927			
Diacavolinia vanutrechti (sp. 2)	[39]	2007-10&11	13°01'N	105°01'W	RV Seward	-	East Pacific Ocean	3	JX183614			3
Diacavolinia vanutrechti (sp. 2)	[39]	2007-10&11	13°01'N	105°01'W	RV Seward	-	East Pacific Ocean	3	JX183616			3
Diacria danae	New	2008-10-30	22°47'S	25°01'W	AMT18	79	South Atlantic Ocean	1	MF048928	MF048980	MF049036	1*
Diacria danae	[35]	-	-	-	CRER 2	-	Caribbean Sea (Yucatan/Belize)	3	KC774075	KC774113		
Diacria danae	[35]	-	-	-	TARA	50	Indian Ocean (North)	3	KC774076			
Diacria danae	[35]	-	-	-	TARA	58	Indian Ocean (Southwest, Mozambique)	3	KC774077			
Diacria danae	[33]	-	-	-	-	-	North Atlantic Ocean (Canary Islands)	2	DQ238001	DQ237987	DQ237968	
Diacria major	New	2008-10-14	27°38'N	37°02'W	AMT18	24	North Atlantic Ocean	1	MF048929	MF048981	MF049037	1*
Diacria major	New	2008-10-16	22°36'N	40°16'W	AMT18	32	North Atlantic Ocean	3	MF048930			1*
Diacria trispinosa	New	2011-01-28	16°22'N	88°02'W	GU1101	77	Caribbean Sea	1	MF048931	MF048982	MF049038	4
Diacria trispinosa	New	2011-01-28	16°22'N	88°02'W	GU1101	77	Caribbean Sea	2	MF048932	MF048983	MF049039	4

Hyalocylis striata	New	2008-10-11	36°01'N	27°44'W	AMT18	15	North Atlantic Ocean	2		MF048984		2
Hyalocylis striata	New	2011-01-16	20°44'N	86°24'W	GU1101	10	Caribbean Sea	1	MF048933			2
Hyalocylis striata	New	2011-01-16	20°44'N	86°24'W	GU1101	10	Caribbean Sea	1		MF048985	MF049040	2
Hyalocylis striata	New	2011-01-16	20°44'N	86°24'W	GU1101	10	Caribbean Sea	2	MF048934		MF049041	2
Hyalocylis striata	New	2012-02-17	24°11'N	109°38'W	MBARI GOC 2012	D333T	Gulf of California	2	MF048935	MF048986	MF049042	1
Hyalocylis striata	New	2012-02-17	24°11'N	109°38'W	MBARI GOC 2012	D333T	Gulf of California	1	MF048936	MF048987	MF049043	
Styliola subula	New	2008-10-28	16°38'S	25°00'W	AMT18	70	South Atlantic Ocean	2		MF048988	MF049044	1
Styliola subula	New	2011-01-19	19°21'N	87°22'W	GU1101	29	Caribbean Sea	1		MF048989	MF049045	3
Styliola subula	[38]	2007-01-24	17°54'N	87°54'W	-	-	Caribbean Sea	1	KF200174			1
Styliola subula	[38]	2007-01-29	18°11'N	87°45'W	-	-	Caribbean Sea	2	KF200175			1
**Limaciniidae**												
Heliconoides inflatus	New	2008-10-21	8°39'N	30°43'W	AMT18	47	North Atlantic Ocean	1*	MF048937		MF049046	1
Heliconoides inflatus	New	2008-10-26	08°50'S	25°00'W	AMT18	63	South Atlantic Ocean	3	MF048938			1
Heliconoides inflatus	New	2008-10-26	08°50'S	25°00'W	AMT18	63	South Atlantic Ocean	2*		MF048990		
Heliconoides inflatus	New	2008-11-04	36°10'S	35°03'W	AMT18	94	South Atlantic Ocean	2*			MF049047	
Heliconoides inflatus	New	2008-11-04	36°10'S	35°03'W	AMT18	94	South Atlantic Ocean	2*	MF048939			1
Heliconoides inflatus	New	2008-11-04	36°10'S	35°03'W	AMT18	94	South Atlantic Ocean	3	MF048940			1
Heliconoides inflatus	New	2010-03-25	42°37'N	18°06'E	KP	4	Mediterranean Sea (Adriatic Sea: Dubrovnik)	1*		MF048991		
Limacina bulimoides	New	2008-10-20	11°49'N	32°49'W	AMT18	44	North Atlantic Ocean	3		MF048992		1
Limacina bulimoides	New	2008-10-31	26°33'S	25°00'W	AMT18	83	South Atlantic Ocean	1*		MF048993	MF049048	
Limacina helicina antarctica	New	2008-11-07	45°13'S	44°37'W	AMT18	101	South Atlantic / Southern Ocean	1	MF048941	MF048994	MF049049	2
Limacina helicina antarctica	New	2008-11-07	45°13'S	44°37'W	AMT18	101	South Atlantic / Southern Ocean	2	MF048942	MF048995		2
Limacina helicina antarctica	[37]	-	-	-	-	-	Southern Ocean (Amundsen Sea)	3	GQ861824			
Limacina helicina antarctica	[37]	-	-	-	-	-	Southern Ocean (Amundsen Sea)	3	GQ861825			
Limacina helicina helicina	[36]	2003-12-03	60°32'N	147°48'W	Alpha-Helix-2003	-	Gulf of Alaska (Prince Williams Sound)	3	FJ876923			
Limacina helicina helicina	[36]	2007-09-04	87°01'N	146°21'W	PS-ARK-23-2	-	Arctic Ocean	3	FJ876924			
Limacina lesueurii	New	2008-10-20	11°49'N	32°49'W	AMT18	44	North Atlantic Ocean	2		MF048996		2
Limacina lesueurii	New	2008-10-20	11°49'N	32°49'W	AMT18	44	North Atlantic Ocean	1		MF048997	MF049050	2
Limacina lesueurii	New	2012-11-09	25°29'S	25°00'W	AMT22	56A	South Atlantic Ocean	2	MF048943			
Limacina lesueurii	New	2012-11-09	25°29'S	25°00'W	AMT22	56A	South Atlantic Ocean	1	MF048944			
Limacina retroversa	New	2004-06-12	56°35'N	31°14'W	MAR-ECO	6	North Atlantic Ocean	1	MF048945	MF048998	MF049051	
Limacina retroversa	New	2004-06-23	50°42'N	27°31'W	MAR-ECO	22	North Atlantic Ocean	2	MF048946	MF048999	MF049052	
Limacina trochiformis	New	2012-10-21	27°36'N	36°22'W	AMT22	19	North Atlantic Ocean	1	MF048947			
Limacina trochiformis	New	2012-11-12	30°10'S	27°54'W	AMT22	60	South Atlantic Ocean	2	MF048948			
Limacina trochiformis	New	2011-01-22	18°52'N	87°17'W	GU1101	34	Caribbean Sea	1		MF049000		2
Limacina trochiformis	New	2011-01-22	18°52'N	87°17'W	GU1101	34	Caribbean Sea	2		MF049001		2
Thielea helicoides	New	2004-06-21	52°45'N	30°30'W	MAR-ECO	20	North Atlantic Ocean	1	MF048949	MF049002		2
Thielea helicoides	New	2004-06-23	50°42'N	27°31'W	MAR-ECO	22	North Atlantic Ocean	2	MF048950	MF049003		

**Thecosomata, Pseudothecosomata**												
**Cymbuliidae**												
Corolla spectabilis	New	2012-11-12	34°15'N	120°09'W	UNOLS CSTC 2012	SB2SB3MOC	Northeast Pacific Ocean (Southern California)	3	MF048951	MF049004		
Corolla spectabilis	New	2012-11-12	34°15'N	120°09'W	UNOLS CSTC 2012	SB2SB3MOC	Northeast Pacific Ocean (Southern California)	2	MF048952	MF049005	MF049053	
Corolla spectabilis	New	2012-02-24	24°22'N	109°13'W	MBARI GOC 2012	D342BW	Gulf of California	3	MF048953			1
Corolla spectabilis	New	2012-02-24	24°22'N	109°13'W	MBARI GOC 2012	D342BW	Gulf of California	1	MF048954	MF049006	MF049054	
Cymbulia sibogae	[36]	2007-11-11	3°13'N	14°04'W	PS-ANT-24-1	-	North Atlantic Ocean	3	FJ876932			
Cymbulia sp.	[35]	-	-	-	ECOSUR	-	Caribbean Sea (Yucatan/Belize)	3		KC774158		
Cymbulia sp.	[35]	-	-	-	TARA	30	Mediterranean Sea (East)	1	KC774090	KC774159		
Gleba cordata	[36]	2006-04-19	25°00'N	59°57'W	RHB0603	-	North Atlantic Ocean	3	FJ876933			
**Desmopteridae**												
Desmopterus sp.	[35]	-	-	-	TARA	40	Gulf of Aden	3		KC774167		
**Peraclidae**												
Peracle bispinosa	[36]	2007-11-11	3°31'N	14°01'W	PS-ANT-24-1	-	North Atlantic Ocean	3	FJ876936			
Peracle bispinosa	[36]	2007-11-11	3°31'N	14°01'W	PS-ANT-24-1	-	North Atlantic Ocean	3	FJ876938			
Peracle reticulata	New	2008-10-12	33°18'N	30°48'W	AMT18	19	North Atlantic Ocean	3		MF049007		1
Peracle reticulata	New	2008-10-29	19°07'S	25°00'W	AMT18	75	South Atlantic Ocean	1		MF049008	MF049055	2
Peracle reticulata	[35]	-	-	-	CRER 2	-	Caribbean Sea (Yucatan/Belize)	1	KC774088			
Peracle reticulata	[35]	-	-	-	ANTEDON	-	Mediterranean Sea (Gulf of Lyon, Cassidaigne)	3	KC774089			
Peracle valdiviae	[36]	2007-11-17	13°25'S	0°39'W	PS-ANT-24-1	-	South Atlantic Ocean	3	FJ876940			
**Gymnosomata**												
**Clionidae**												
Clione limacina antarctica	[34]	2009-02-20	61°46'S	45°27'W	Nerida Wilson	-	Southern Ocean (South Orkney Islands)	1	CEF048	CEF048	CEF048	
Clione limacina	New	2012-07-17	36°42'N	122°03'N	MBARI MW 2012	D416T.11	Northeast Pacific Ocean (Northern California)	1	MF048955	MF049009	MF049056	1
Clione limacina	New	2012-07-17	36°42'N	122°03'N	MBARI MW 2012	D416T.15	Northeast Pacific Ocean (Northern California)	2	MF048956	MF049010	MF049057	
Clione limacina	[36]	2006-04-13	33°31'N	69°58'W	RHB0603	-	North Atlantic Ocean	3	FJ876941			
Clione limacina	[36]	2005-07-11	74°35'N	151°56'W	Healy 05/2	-	Arctic Ocean (Canadian Basin)	3	FJ876944			
Thliptodon sp.	New	2012-02-24	24°22'N	109°13'W	MBARI GOC 2012	D342BW	Gulf of California	1	MF048957	MF049011	MF049058	1
Thliptodon sp.	New	2013-06-09	36°35'N	122°31'W	MBARI MW 2013	D484D3	Northeast Pacific Ocean (Northern California)	2	MF048958	MF049012	MF049059	1
Thliptodon diaphanus	[36]	2006-04-20	24°50'N	60°27'W	RHB0603	-	North Atlantic Ocean	3	FJ876950			
**Cliopsidae**												
Cliopsis krohni	New	2013-06-09	36°34'N	122°31'W	MBARI MW 2013	D484T	Northeast Pacific Ocean (Northern California)	1	MF048959	MF049013	MF049060	1
Cliopsis krohni	New	2013-06-09	36°34'N	122°31'W	MBARI MW 2013	D484T	Northeast Pacific Ocean (Northern California)	3	MF048960			1
Cliopsis krohni	New	2013-08-30	39°56'N	67°17'W	NOAA PC201205	NWAT1	Northwest Atlantic Ocean	1	MF048961	MF049014	MF049061	1
Cliopsis krohni	New	2012-09-04	39°57'N	67°27'W	NOAA PC201205	NWAT20	Northwest Atlantic Ocean	2	MF048962	MF049015	MF049062	1
**Notobranchaeidae**												
Notobranchaea macdonaldi	New	2013-03-22	36°41'N	122°10'W	MBARI MW 2013	D448T	Northeast Pacific Ocean (Northern California)	1	MF048963	MF049016		1

**Pneumodermatidae**												
Pneumoderma atlantica	[33]	-	-	-	-	-	North Atlantic / Indian / Pacific Ocean (USA/Australia)	1	DQ238003	DQ237989	DQ237970	
Pneumoderma violaceum	[36]	2007-11-08	11°23'N	20°21'W	PS-ANT-24-1	-	North Atlantic Ocean	3	FJ876945			
Pneumodermopsis macrochira	[36]	2006-04-16	29°52'N	70°05'W	RHB0603	-	North Atlantic Ocean	3	FJ876946			
Pneumodermopsis sp.	New	2012-02-12	23°42'N	108°49'W	MBARI GOC 2012	D339T	Gulf of California	1	MF048964	MF049017	MF049063	1
Pneumodermopsis sp.	New	2012-02-12	23°42'N	108°49'W	MBARI GOC 2012	D339T	Gulf of California	2	MF048965	MF049018	MF049064	
Schizobrachium polycotylum	[36]	2007-11-11	3°31'N	14°01'W	PS-ANT-24-1	-	North Atlantic Ocean	3	FJ876949			
Spongiobranchaea australis	[33]	-	-	-	-	-	Southern Ocean (Scotia Arc)	1	DQ238002	DQ237988	DQ237969	
**Outgroup taxa**												
**Cephalaspidea, Bulloidea, Bullidae**												
Bulla striata	[33]	-	-	-	-	-	North Atlantic Ocean (Bermuda)	1	DQ238005	AY427477	AY427512	
**Anaspidea, Aplysioidea, Aplysiidae**												
Aplysia californica	[33]	-	-	-	-	-	Atlantic Ocean	1	AF077759	AY026366	AY039804	
